# Pharmacological Inhibition of the NLRP3 Inflammasome: Structure, Molecular Activation, and Inhibitor-NLRP3 Interaction

**DOI:** 10.1124/pharmrev.122.000629

**Published:** 2023-05

**Authors:** Qiang Ma

**Affiliations:** Receptor Biology Laboratory, Toxicology and Molecular Biology Branch, Health Effects Laboratory Division, National Institute for Occupational Safety and Health, Centers for Disease Control and Prevention, Morgantown, West Virginia

## Abstract

The nucleotide-binding, oligomerization domain-like receptor family pyrin domain containing 3 (NLRP3) inflammasome is a multiprotein complex that combines sensing, regulation, and effector functions to regulate inflammation in health and disease. NLRP3 is activated by a diverse range of inflammation-instigating signals known as pathogen associated molecular patterns and danger associated molecular patterns. Upon activation, NLRP3 oligomerizes and recruits partner proteins to form a supramolecular platform to process the maturation and release of interleukin (IL)-1*β*, IL-18, and gasdermin D, major mediators of inflammation and inflammatory cell death termed pyroptosis. The NLRP3 inflammasome has been implicated in the pathogenesis of a wide range of disease conditions, including chronic inflammatory disease that are associated with lifestyle and dietary changes, aging, and environmental exposures, and have become the leading cause of death worldwide. Pharmacological targeting of NLRP3 and signaling demonstrated promising efficacy in ameliorating a list of disease conditions in animal models. These findings underscore the potential and importance of NLRP3 as a druggable target for treating a range of diseases. In this review, recent progress in understanding the structure and mechanism of action of the NLRP3 inflammasome is discussed with focus on pharmacological inhibition of NLRP3 by small molecule inhibitors. New structural and mechanistic insights into NLRP3 activation and inhibitor-NLRP3 interactions would aid in the rational design and pharmacological evaluation of NLRP3 inhibitors for treatment of human disease.

**Significance Statement:**

The NLRP3 inflammasome plays central role in innate immune sensing and control of inflammation. Pharmacological inhibition of NLRP3 demonstrated promising efficacy in a range of diseases in animal models. Recent elucidation of the structure and inhibitor binding of NLRP3 generated new insights into its mode of action and inhibitor-NLRP3 interaction at molecular levels, providing new framework for developing small chemical inhibitors of NLRP3 with improved efficacy and specificity against chronic disease that has become major health concerns worldwide.

## Graphical abstract

**Figure:**
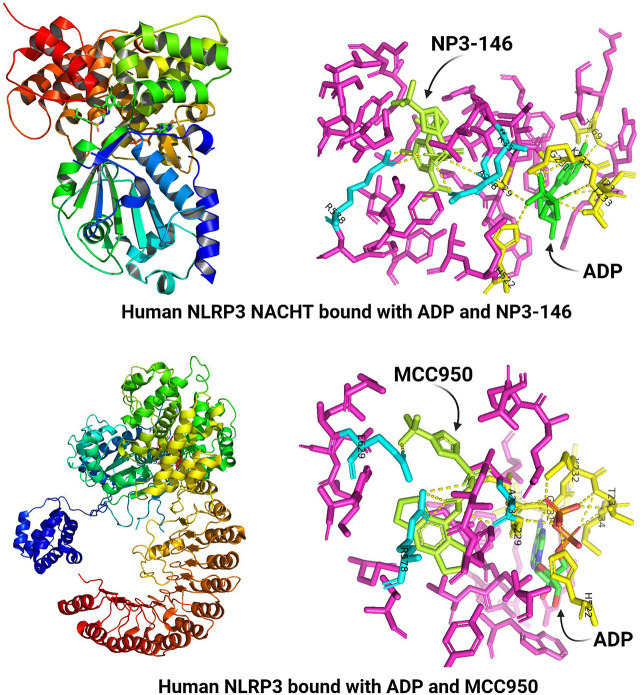

## I. Introduction

Pharmacologists and the drug industry have long been interested in developing remedies to treat chronic disease. Chronic, noncommunicable diseases that are associated with lifestyle, dietary, social, and environmental factors are of particular concern, as they have become the leading cause of mortality and disability in the world today. These include chronic cardiovascular disease, cancer, chronic metabolic disorders, autoimmune conditions, and neurodegenerative illnesses. Notwithstanding, this task has proven to be very challenging, due in part to the complexity of pathogenesis and lack of effective druggable targets of chronic disease collectively and individually.

Chronic inflammation is a common component of pathogenesis of chronic disease. Moreover, chronic inflammation-driven illnesses have become the most significant cause of all death combined worldwide (GBD 2017 Causes of Death Collaborators, 2018; Furman et al., 2019). From a mechanistic point of view, the initiation and propagation of chronic inflammation in chronic disease are mediated and regulated by the same immune strategies that help defend the body against pathogens. Central to this process is the timely recognition of inflammation-instigating signals by the immune system. In this respect, the nucleotide-binding, oligomerization domain (NOD)-like receptor (NLR) family pyrin domain containing 3 (NLRP3) inflammasome has received particular attention. The NLRP3 inflammasome brings together innate immune sensing, regulation, and effector functions to initiate and control inflammatory responses in a wide range of disease conditions, including chronic inflammatory illnesses (Schroder and Tschopp, 2010; Swanson et al., 2019). Fittingly, there has been a rapidly growing interest in developing drugs that modulate the activity of the NLRP3 inflammasome for treating diseases in humans, and some have shown efficacy in animal models and clinical trials in recent years (Mangan et al., 2018).

A major obstacle that has hindered the therapeutic targeting of NLRP3 is the lack of a good understanding of the structure and mechanism of action of NLRP3. In this connection, several studies have provided new insights into the structure, activation, and signaling of NLRP3. In particular, the three-dimensional structure of inactive NLRP3 in complex with an activating protein has been revealed at a resolution of 3.8 Å by cryo-EM (Sharif et al., 2019). A structure of NLRP3 domain present in NAIP, CIITA, HET-E, and TP1 (NACHT) bound with an analog of MCC950—a potent and specific inhibitor of NLRP3 initially named cytokine release inhibitory drug (CRID) 3 or CP-456,773—was obtained by crystallography at a resolution of 2.8 Å (Dekker et al., 2021). Binding of MCC950 and its analogs to NLRP3 NACHT was also demonstrated by biophysical and biochemical means, revealing that MCC950 likely interacts with the nucleotide-binding motives within the NACHT domain to obstruct ATP/ADP binding and/or ATP hydrolysis (Coll et al., 2019; Tapia-Abellán et al., 2019; Vande Walle et al., 2019). Recent studies uncovered the formation of ring-like oligomeric structures of inactive NLRP3 with or without MCC950; moreover, these ring structures are associated with intracellular membrane structures and membrane trafficking for docking and activation (Andreeva et al., 2021; Hochheiser et al., 2022b; Ohto et al., 2022). Homotypic interactions among pyrin domains (PYD) of activated NLRP3 and its adaptor protein associated speck-like protein containing a caspase-recruitment and activation domain (ASC) orchestrate the formation of NLRP3 PYD nucleation seeds that in turn directs the prion-like, directional oligomerization of ASC into filamentous bundles leading to speck formation (Hochheiser et al., 2022a). Together, these findings suggest new structural and mechanistic frameworks that can be used to facilitate future investigation of the function and mechanism of action of NLRP3 and its inhibition by small molecule inhibitors. From this prospective, I discuss recent advances in our understanding of the structure and molecular activation of NLRP3 with highlight on inhibitor-NLRP3 interaction in pharmacological targeting of the NLRP3 inflammasome for treatment of inflammatory disease.

## II. NLRP3 Inflammasome in Health and Disease

### A. NLRP3 as a Sensor of Infection and Injury

Inflammation reflects the adaptation of multicellular organisms to restore homeostasis after infection and injury. Inflammation as a whole is largely mediated through the innate immune system (Barton, 2008). In this process, activated innate immune cells coordinate with increased blood flow and secretion of soluble factors to mediate chemoattraction, phagocytosis, killing of microbes, removal of tissue debris, and repair of damaged tissue (Nathan, 2002). At the core of these reactions is the recognition of signals derived from pathogens termed pathogen associated molecular patterns (PAMPs) and from the host known as danger (damage) associated molecular patterns (DAMPs). Detection of PAMPs and DAMPs is mediated through germline-encoded pattern recognition receptors consisting of membrane-bound receptors, such as the toll-like receptors (TLRs), and intracellular receptors, such as inflammasomes (Janeway, 1989; Janeway and Medzhitov, 2002; Tschopp et al., 2003). TLRs scan extracellular PAMPs and transduce the signals through pathways like nuclear factor kappa B (NF-*κ*B) to regulate inflammation (Medzhitov and Janeway, 1997). Inflammasomes detect intracellular PAMPs and sterile DAMPs but are much less well understood for their mechanism of activation and function compared with their membrane counterparts.

Initially coined by Jürg Tschopp and associates, the term inflammasome denotes intracellular supramolecular protein complexes that assemble to mediate the activation of proinflammatory caspase 1 (Casp1) and the maturation and secretion of IL 1*β* in response to pathogens and sterile stimuli (Martinon et al., 2002; Schroder and Tschopp, 2010). An inflammasome typically consists of a sensor protein that aggregates upon activation and recruits two additional proteins, ASC and pro-caspase 1. The NLR family of inflammasomes use sensor proteins, such as NLRP3 and NLR family CARD domain-containing protein 4 (NLRC4), that each contains an NTP-binding NACHT domain, whereas the absent in melanoma 2 (AIM2)-like receptor family of inflammasomes, such as AIM2 and IFI16, do not have an NACHT domain (Man and Kanneganti, 2015). In both types of inflammasomes, recruited pro-caspase 1 cleaves itself into an active enzyme, which in turn cleaves the precursors of proinflammatory cytokines IL-1*β* and IL-18 into mature cytokines. Secreted IL-1*β* and IL-18 initiate and amplify inflammatory responses in a context-dependent manner (Swanson et al., 2019). In addition to the IL-1 family of cytokines, Casp1 cleaves the intracellular protein gasdermin D (GSDMD) to result in the formation of pores in the cytoplasmic membrane, followed by swelling and lysis of the cell known as inflammatory cell death or pyroptosis (Liu et al., 2016). Release of other inflammatory mediators, such as IL-1*α* and high mobility group box 1 protein, further enhances the inflammatory response.

The list of known and potential substrates of inflammasomal Casp1 continues to grow. By using targeted peptidecentric proteomics, about 1022 proteins were identified as having Casp1 processing sites. Among the proteins, 20 were shown to be specifically cleaved by Casp1 in vitro and Casp7 was validated as a Casp1 substrate using knockout macrophages lacking Casp1 or ASC (Lamkanfi et al., 2008). Casp7 is known to be associated with apoptosis by cleaving substrates such as the DNA damage sensor protein poly(ADP-ribose) polymerase 1 (PARP1). Cleavage of PARP1 by Casp3 or Casp7 is a hallmark of apoptosis. Activation of the NLRP3 inflammasome induced the cleavage of PARP1 via Casp1 and Casp7, implicating PARP1 activation in inflammasomal pyroptosis independently of apoptosis (Malireddi et al., 2010). Activation of PARP1 by Casp7 can occur at the promoters of a subset of NF-*κ*B target genes, providing a mechanism by which proinflammatory gene expression is regulated upon activation of the NLRP3 inflammasome (Erener et al., 2012).

**Fig. 1:**
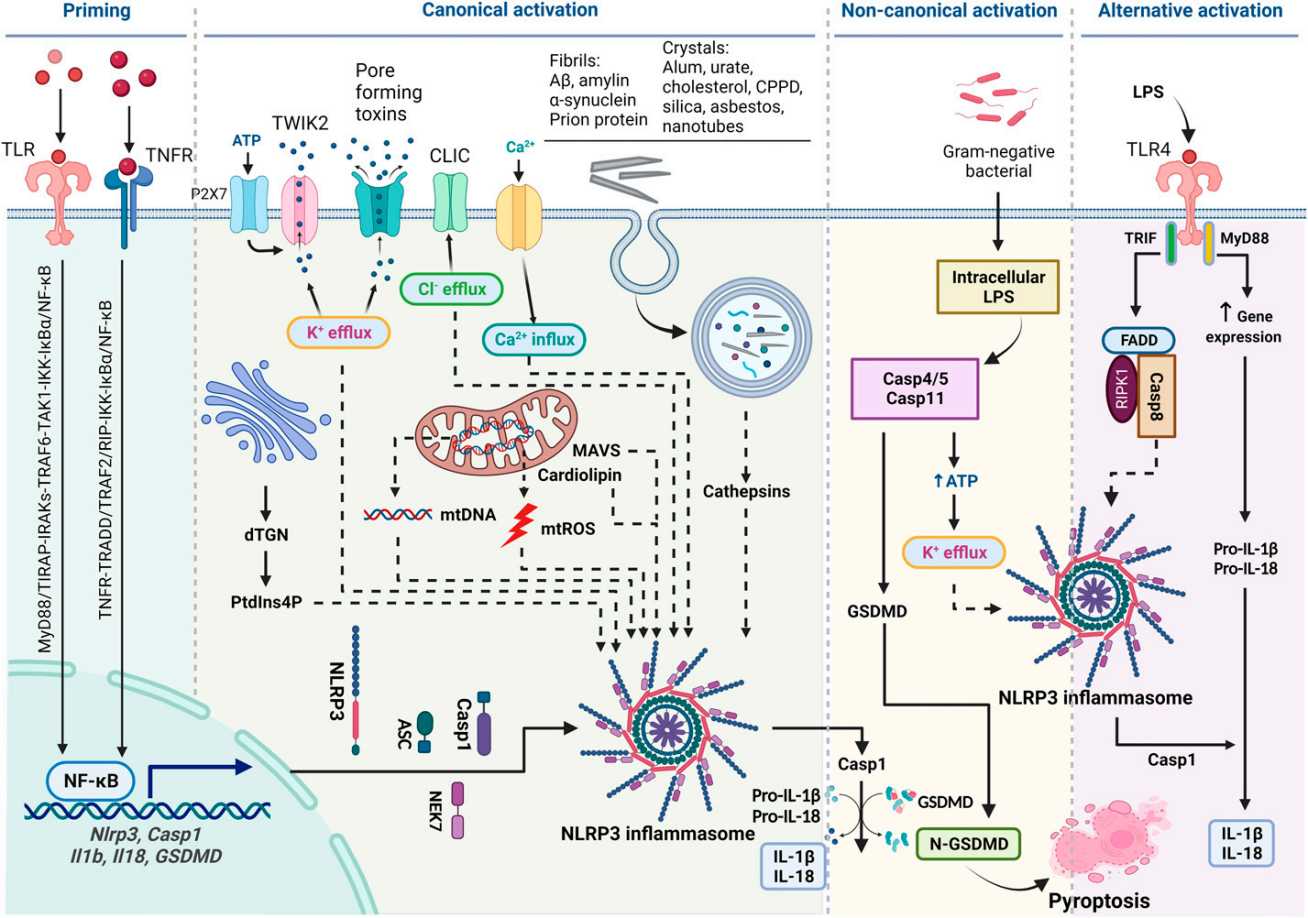
Diagram of activation of NLPR3 inflammasome. Priming is induced by signal 1, such as LPS and TNF-*α*, that upregulates the transcription of genes encoding NLRP3 inflammasome components by activating their membrane-bound pattern recognition receptors. Canonical activation of NLRP3 inflammasome is elicited by signal 2 including PAMPs, such as nigericin, viral RNA, and MDP, and DAMPs, such as extracellular ATP, mtDNA, and mtROS, and particles and crystals. Activation involves multiple signaling events including K^+^ efflux, Ca^2+^ flux, Cl^–^efflux, lysosomal disruption, mtROS production, and release of oxidized mtDNA. Activation may also involve docking of NLRP3 at mitochondrial outer membrane via mitochondrial antiviral signaling protein or cardiolipin and at dispersed dTGN through PtdIns4P. Formation of NLRP3 inflammasome includes binding of NLRP3 with NEK7, oligomerization of NLRP3, assembly of ASCs into fibrils, and recruitment and activation of Casp1. Casp1 in turn activates Pro-IL-1*β* and Pro-IL-18 and GSDMD by proteolytic cleavage of the proteins. IL-1*β* and IL-18 are secreted to promote proinflammatory responses, whereas N-GSDMD forms pores in the plasma membrane to result in pyroptosis of the cell. Noncanonical activation of NLRP3 inflammasome is induced by gram-negative bacteria. Release of LPS from engulfed bacteria into the cytoplasm activates human Casp4/5 or mouse Casp11, which cleaves GSDMD to induce pyroptosis and indirectly activates the NLRP3 inflammasome to activate Casp1 and IL-1*β* and IL-18. Noncanonical activation does not require priming as Casp4 is present at a high level. The alternative pathway of activation is elicited by TLR4 agonists like LPS, which activates the TLR4-TRIF-RIPK1-FADD-Casp8 signaling. Casp8 activates the NLRP3 inflammasome but does not require K^+^ efflux, ASC speck formation, or pyroptosis.

Many inflammasomal sensor proteins are activated by specific signals from pathogens. For instance, NLRC4 is activated by bacterial flagellin or T3SS, a component of the Salmonella type III secretion system, whereas AIM2 senses cytoplasmic double-stranded DNAs that are generated from DNA damage (Schroder et al., 2009; Duncan and Canna, 2018). The NLRP3 inflammasome is unique in that it is activated by a broad range of structurally diverse signals, ranging from microbial components, such as nigericin, muramyl dipeptide, and double- or single-stranded RNA, to host cell molecules, such as extracellular ATP and mitochondrial reactive oxygen species (ROS) (Fig. 1). Notably, a variety of particulates including metabolite crystals and protein aggregates produced endogenously, as well as mineral particles and nanoparticles encountered from exogenous exposures, activate NLRP3 (Schroder and Tschopp, 2010). This broad sensitivity of NLRP3 to inflammation-stimulating signals enables the inflammasome to sense and initiate responses in a wide variety of physiologic and disease conditions.

### B. The NLRP3 Inflammasome Connects to Chronic Disease by Chronic Inflammation

Aberrant activation and function of the NLRP3 inflammasome have been implicated in the development of a broad spectrum of disease conditions. These include autoinflammatory diseases known as cryopyrin-associated periodic syndromes (CAPS) that are caused by hereditary gain-of-function mutations of the NLRP3 gene; infectious illnesses that display prominent inflammatory lesions; and chronic diseases that are marked by chronic inflammation elicited by sterile danger signals derived from metabolic abnormality, accumulation of crystals and aggregates, neurodegeneration, and oncogenic and metastatic processes (Mangan et al., 2018). In this connection, the study on the NLRP3 inflammasome has led to a rapid advancement in our understanding of innate immune sensing and effector responses in health and disease at molecular levels during the past twenty years (Hoffman et al., 2001; Guo et al., 2015; Sharma and Kanneganti, 2016; Mangan et al., 2018; Kelley et al., 2019; Swanson et al., 2019; Zahid et al., 2019; McKee and Coll, 2020).

Chronic disease commonly exhibits inflammatory conditions known as systemic chronic inflammation, a state of low-grade, systemic, and noninfective inflammation (Furman et al., 2019). Systemic chronic inflammation is believed to be brought about by a variety of sterile danger signals that are produced and accumulated over time from diseased tissue. This process is exacerbated by many biologic, social, and environmental factors that impede the resolution of inflammation. Under a physiologic condition, an inflammatory response is presented as a temporally restricted upregulation of immune, vascular, and tissue activities that take place in the presence of an inflammation-stimulating signal but would resolve as the instigator is eliminated and homeostasis is retained. Under chronic conditions where a stimulus persists or the resolution of acute inflammation is obstructed or both, inflammation persists leading to chronic inflammation. Systemic chronic inflammation can cause major alterations in the structure and function of involved tissue and organs to disrupt the normal physiology and breakdown immune tolerance, all of which increase the risk for noncommunicable, chronic disease in susceptible individuals.

Chronic disease–associated inflammation is initiated and regulated by the same innate immune mechanisms that mediate the protective, acute inflammatory response against infection. In this regard, the NLRP3 inflammasome has received notable attention for its role in sensing a wide range of chronic inflammation-associated DAMPs. Aberrant functions of the NLRP3 inflammasome have been implicated in the pathogenesis and exacerbation of a variety of chronic conditions. In these cases, the NLRP3 inflammasome elicits prolonged inflammation locally and systemically in response to sterile, host-derived signals associated with aging, overnutrition, and physical inactivity that are typically recurrent or chronic. NLRP3 is also activated by nonmicrobial environmental toxicants, including mineral crystals and fibers and nanoparticles that cause inflammation, fibrosis, and cancer in the lung and the pleural space, exemplified by pneumoconiosis, lung cancer, and mesothelioma resulting from inhalation of crystalline silica and asbestos. These findings support an important role of the NLRP3 inflammasome in the connection between chronic inflammation and chronic disease.

### C. NLRP3 Inflammasome-Associated Disease

Disease and pathologic conditions that the NLRP3 inflammasome has been associated with are summarized in Table 1. These include autoinflammatory disease, microbial infection, metabolic disease, common chronic inflammatory and autoimmune illnesses, endogenous and environmental particulates-driven pathologic conditions, and cancer and metastasis.

**TABLE 1:** NLRP3 inflammasome-driven disease

Associated Disease	Activator and Mechanism	Disease and Pathologic Feature	Genetic and Pharmacologic Intervention	Ref*^a^*
Autoinflammatory disease				
FCAS MWS NOMID	Gain-of-function mutation in NACHT to disrupt autoinhibition of NLRP3	Fever, neutrophilia, multiorgan inflammation (FCAS, MWS, NOMID). Hearing loss (MWS, NOMID). CNS inflammation (NOMID)	Autoinflammation in mice expressing CAPS variants. Treatment by inhibition of IL-1 and NLRP3	1
Infection				
IAV	Viral PAMPs and DAMPs IAV M2 protein	Hyperinflammation	MCC950 protects juvenile mice from IAV hyperinflammation	2
SARS-Cov-2	Viral PAMPs and DAMPs	Airway and lung inflammation in mild and severe SARS-Cov-2 patients	Activation of NLRP3 inflammasome in PBMCs and autopsy tissues	3
Metabolic disease and aging				
Obesity, type 2 diabetes	Ceramide, fatty acids, and obesity-associated DAMPs	Obesity, low-grade inflammation, fatty liver, insulin resistance	Ablation of NLRP3 ↓ obesity-induced inflammation and insulin resistance	4
Aging	Low-grade inflammation	Systemic low-grade inflammation and multiple aging phenotypes including ↓ lipolysis and ↑ SASP	Ablation of NLRP3 inflammasome protected from multiple, aging-driven phenotypes	5
Common inflammatory/immune pathologic condition				
Asthma	Allergic immune signals, nonallergic stimulants	Allergic airway inflammation in mice induced by ovalbumin or house dust mite extract	Ablation of the NLRP3 inflammasome pathway ↓ allergic airway inflammation. MCC950 ↓ airway inflammation	6
IBD	Inflammatory DAMPs	DSS-induced colitis in mice. DNBS-induced colitis in rats	Ablation of NLRP3 ↓ severity of mouse colitis. Inhibition of NLRP3 by INF39 ↓ severity of rat colitis	7
NAFLD	Fatty acid, cholesterol	Choline deficient amino acid defined- or methionine/choline-deficient diet induced NAFLD in mice	Ablation of NLRP3 protected mice from induced NAFLD and fibrosis. MCC950 ↓ induced NASH and liver scarring	8
RA	Inflammatory DAMPs	Spontaneous erosive polyarthritis in A20(myol-KO) mice	Ablation of NLRP3 protected against rheumatoid arthritis-associated inflammation and cartilage destruction	9
MS, EAE	DAMPs	Autoimmune encephalomyelitis	Ablation of NLRP3 led to resistance to EAE. MCC950 ↓ the severity of EAE	10
Endogenous particulates-associated pathology				
Gout and pseudogout	MSU or CPPD crystal Phagocytosis and lysosomal disruption	Gout and pseudogout arthritis. Urate or CPPD crystal-induced inflammation	Deficiency of NLRP3 inflammasome ↓ urate crystal-induced inflammation. *β*-Hydroxybutyrate ↓ gouty flares by ↓ NLRP3 activation	11
Atherosclerosis	Cholesterol crystal Phagocytosis and lysosomal disruption	Diet- and genetic-induced atherosclerosis; low-level inflammation	Ablation of NLRP3 and other genes ↓ atherosclerotic lesions in mice. MCC950 ↓ atherosclerotic lesions	12
AD	A*β* fibril Released by dead neurons; phagocytosed by microglia	Senile plaques in brain neurons. Cerebral neuroinflammation. Mouse APP/PS1 model	Activation of NLRP3 inflammasome by A*β* fibril. Ablation of NLRP3 improves memory and clearance. Fenamate NSAID and MCC950 protects against AD in mice	13
PD	*α*Syn fibril Released by dead neurons; phagocytosed by microglia	LB aggregates in dopaminergic neurons of *substantial nigra* Loss of dopaminergic neurons	Activation of NLRP3 inflammasome by aSyn fibrils	14
Exogenous particulates-associated pathology				
Immunization	Aluminum	Phagocytosis and lysosome disruption	Enhancing immunization effect as immunization adjuvant	15
Silicosis, lung cancer	Crystalline silica Phagocytosis. lysosomal disruption. ↑ROS. TXNIP-NLRP3 binding	Acute and chronic inflammation, interstitial fibrosis, granuloma formation, and cancer in the lung	Activation of NLRP3 inflammasome in macrophages by silica in vitro and in the lung	16
Asbestosis, mesothelioma	Asbestos Phagocytosis. lysosomal disruption. ↑ROS	Acute and chronic inflammation, interstitial fibrosis, granuloma formation, cancer in the lung, and mesothelioma in the pleura	NLRP3−/− mice show reduced lung inflammation upon inhalation of asbestos	17
Cancer and metastasis				
Tumor and metastasis	Low-grade inflammation	MCA-induced lung cancer; metastasis	Ablation of NLRP3 ↓ tumor burden by ↑ NK cell responses	18
Anti-tumor vaccination	Low-grade inflammation	Dendritic cell vaccination against melanoma	Ablation of NLRP3 ↑vaccination effect by ↓ tumor MDSCs	19

*^a^*Reference cited: 1. Aksentijevich et al., 2002; Broderick et al., 2015; Brydges et al., 2013; de Torre-Minguela et al., 2017; Hoffman et al., 2001; Mangan et al., 2018; Masters et al., 2009; Sharif et al., 2019; 2. Coates et al., 2017; Tate et al., 2016; 3. Rodrigues et al., 2021; 4. Ralston et al., 2017; Vandanmagsar et al., 2011; 5. Acosta et al., 2013; Camell et al., 2017; Youm et al., 2013; 6. Besnard et al., 2011; Primiano et al., 2016; 7. Bauer et al., 2010; Cocco et al., 2017; 8. Mridha et al., 2017; Wree et al., 2014; 9. Vande Walle et al., 2014; 10. Coll et al., 2015; Furlan et al., 1999; Huang et al., 2004; Inoue et al., 2012; Matsuki et al., 2006; 11. Goldberg et al., 2017; Martinon et al., 2006; 12. Duewell et al., 2010; Karasawa and Takahashi, 2017; Rajamäki et al., 2010; van der Heijden et al., 2017; 13. Daniels et al., 2016; Dempsey et al., 2017; Halle et al., 2008; Heneka et al., 2013; 14. Codolo et al., 2013; 15. Eisenbarth et al., 2008; Hornung et al., 2008; 16. Cassel et al., 2008; Dostert et al., 2008; Hornung et al., 2008; Peeters et al., 2014; 17. Dostert et al., 2008; 18. Chow et al., 2012; Moossavi et al., 2018; 19. van Deventer et al., 2010.

A*β*, amyloid beta; AD, Alzheimer’s disease; *α*Syn, *α*-synuclein; CPPD, calcium pyrophosphate dihydrate; DNBS, 2,4-dinitrobenzenesulfonic acid; DSS, dextran sodium sulfate; EAE, experimental autoimmune encephalomyelitis; FCAS, familial cold auto-inflammatory syndrome; IBD, irritable bowel disease; LB, Lewy body; MCA, methylcholanthrene; MDSC, myeloid-derived suppressor cell; MS, multiple sclerosis; MSU, monosodium urate; NAFLD, nonalcoholic fatty liver disease; NK, natural killer; NSAID, nonsteroidal anti-inflammatory drug; PBMC, peripheral blood mononuclear cell; PD, Parkinson’s disease; RA, rheumatoid arthritis; ROS, reactive oxygen species; SASP, senescence-associated secretory phenotype.

#### 1. Autoinflammatory Disease

More than 200 mutations in the NLRP3 gene associated with CAPS have been identified (Sarrauste de Menthière et al., 2003; Booshehri and Hoffman, 2019; Samson et al., 2020). These mutations produce gain-of-function NLRP3 proteins that form functional inflammasome in the absence of an apparent activating signal when overexpressed, thereby causing autoinflammatory disease (Masters et al., 2009). CAPS include the familial cold autoinflammatory syndrome (FCAS), Muckle-Wells syndrome (MWS), and neonatal-onset multisystem inflammatory disorder (NOMID) (Broderick et al., 2015). Clinically, CAPS are characterized by fever, neutrophilia, and multiorgan inflammation. Hearing loss occurs in MWS and NOMID, whereas central nervous system (CNS) inflammation is observed in NOMID. Transgenic mice expressing CAPS variants of NLRP3 developed autoinflammatory lesions, which were dependent on the presence of functional ASC, Casp1, and IL-1*β* (Brydges et al., 2013). Treatment with drugs that target IL-1*β* (i.e., canakinumab and rilonacept) or its receptor (i.e., anakinra) effectively ameliorated inflammatory symptoms in patients with CAPS (Jesus and Goldbach-Mansky, 2014).

#### 2. Infection

The NLRP3 inflammasome is activated in several microbial infections. The influenza A virus (IAV) causes life-threatening lower respiratory tract infections in children. The NLRP3 inflammasome is activated in IAV infection by the IAV matrix 2 proton channel protein. Inhibition of the NLRP3 inflammasome with MCC950 in a mouse model of juvenile IAV infection was shown to improve the survival of mice accompanied by reduction of NLRP3 and decreased secretion of IL-18 into the alveolar fluid (Coates et al., 2017). Severe cases of COVID-19 are often presented with strong inflammation in the lungs of patients leading to respiratory failure. The NLRP3 inflammasome is activated during SARS-CoV-2 infection and is active in COVID-19 patients and in autopsy tissues. Moreover, higher levels of inflammasome products, such as IL-18 and Casp1p20, correlated with the severity of the disease and poor clinical outcomes, supporting a role of the NLRP3 inflammasome in COVID-19 pathogenesis and progression (Rodrigues et al., 2021).

#### 3. Metabolic Disease and Aging

The prevalence of metabolic disease is rising at a fast pace across the globe, which, in large part, is attributable to overnutrition, sedentary lifestyle, and aging of the modern society. Under these conditions, extra energy from consumption of excess food is stored as fat, giving rise to obesity, insulin resistance, atherosclerosis, and hypertension. Products of metabolic dysbiosis, such as certain free fatty acids, cholesterol crystals, advanced glycation end products, and protein aggregates, accumulate in tissues and serve as DAMPs. These DAMPs activate the NLRP3 inflammasome, which stimulates chronic, low-grade, sterile systemic inflammation. Chronic inflammation in turn fuels the development of metabolic diseases, such as type 2 diabetes, non-alcoholic steatohepatitis (NASH), chronic kidney disease, and aging-associated neurodegeneration.

In obese and diabetic individuals, NLRP3 was found to be activated by lipotoxicity-associated increase in intracellular ceramide in macrophages and adipose tissue. Caloric restriction and exercise-mediated weight loss in the individuals caused a reduction of NLRP3 expression in adipose tissue, along with decreased inflammation and improved insulin sensitivity (Vandanmagsar et al., 2011). In the same study, loss of Nlrp3 in obese mice reduced IL-18 and adipose tissue IFN-*γ* expression, supporting a role of NLRP3 in fatty tissue inflammation in obese mice. Systemic low-grade inflammation promotes age-related degeneration. In a mouse study, knockout of NLRP3 protected mice from age-related increases in innate immune activities and gliosis, accompanied by improved glycemic control, attenuated bone loss and thymic demise, and improvement in cognitive functions and motor performances in aged mice (Youm et al., 2013).

#### 4. Common Inflammatory and Autoimmune Disease

Asthma is a common inflammatory condition of the lung airway characterized by recurrent narrowing, swelling, and mucus secretion of the airway causing difficult breathing. Asthmatic attacks are triggered by exposure to allergens and some nonallergic stimulants. In a mouse model of asthma induced by ovalbumin, the NLRP3 inflammasome was activated, leading to increased IL-1 production, which was critical for the induction of a T helper 2 type of inflammatory allergic response in the airway (Besnard et al., 2011). In a mouse model of asthma induced by house dust mite extract, inhibition of the NLRP3 inflammasome with MCC950 (i.e., CP-456,773) effectively reduced airway inflammation upon acute exposure to house dust mite extract (Primiano et al., 2016).

IL-1*β* and IL-18 are known to play a central role in the pathogenesis of irritable bowel disease. In a mouse model of colitis induced by dextran sodium sulfate, the NLRP3 inflammasome was activated and shown to be required for production of IL-1*β* and IL-18, and for dextran sodium sulfate-induced colitis (Bauer et al., 2010). In a rat model, inhibition of the NLRP3 inflammasome by INF39, an acrylate derivative and irreversible inhibitor of NLRP3, decreased IL-1*β* secretion and alleviated colitis induced by 2,4-dinitrobenzenesulfinic acid in rats (Cocco et al., 2017).

In murine models of NASH, cholesterol crystals were shown to activate NLRP3 in the liver. Inhibition of the NLRP3 inflammasome with MCC950 partially reversed the liver inflammation and liver scarring, particularly in obese diabetic mice that mimics NASH in humans (Mridha et al., 2017). In a separate study, NLRP3 inflammasome gain-of-function in mice resulted in early and severe onset of diet-induced NASH, whereas the loss of its function in mice provided protection from NASH. Moreover, patients with severe nonalcoholic fatty liver disease showed increased levels of NLRP3 and IL-1*β* mRNA that correlated with the expression of COL1A1, a marker of liver fibrosis (Wree et al., 2014).

Rheumatoid arthritis is a common chronic autoimmune inflammatory disease characterized by multijoint inflammation and cartilage destruction. IL-1 is recognized as an important mediator of cartilage destruction. Deletion of the rheumatoid arthritis susceptibility gene A20/Tnfaip3 in mice [i.e., the A20(myel-KO) mice] induced spontaneous rheumatoid arthritis. A20-deficiency in macrophages increased NLRP3 inflammasome-mediated Casp1 activation, pyroptosis, and IL-1*β* secretion in the presence of soluble and crystalline NLRP3 activators. Genetic experiments showed that NLRP3 inflammasome activation was required for the development of rheumatoid arthritis and cartilage destruction in the mice (Vande Walle et al., 2014).

Multiple sclerosis is an autoimmune inflammatory illness of the CNS caused by inflammation and demyelination of nerves. Expression of Casp1 and IL-18 was found to be increased in the peripheral blood mononuclear cells of patients with multiple sclerosis (Huang et al., 2004). Experimental autoimmune encephalomyelitis reflects the neuroimmune response to priming with CNS-restricted antigens and is a useful model for some aspects of multiple sclerosis. Progression of encephalomyelitis in the spinal cord was accompanied by increased expression of NLRP3, whereas loss of NLRP3, Casp1, ASC, or IL-1*β* in mice led to resistance to encephalomyelitis (Furlan et al., 1999; Matsuki et al., 2006; Inoue et al., 2012). Treatment of mice with MCC950 blocked NLRP3 activation at a nanomolar concentration, which reduced IL-1*β* production and attenuated the severity of experimental encephalomyelitis in vivo (Coll et al., 2015).

#### 5. Endogenous Particulate-Associated Pathology

A prominent feature in the action of NLRP3 derives from the observation that the NLRP3 inflammasome can be induced by a variety of particles and crystals. These particulates are largely micro and nano in size but vary tremendously in their source, shape, composition, surface property, tissue distribution, and interaction with the immune system. Upon accumulation, these particulates typically elicit chronic inflammation in the body, leading to various chronic pathologic conditions. Endogenous particulates result from the excess production and accumulation of metabolites, such as waste metabolites, cholesterol, and misfolded proteins, in tissues to induce chronic inflammation. Gout and pseudogout are caused by the production and deposition of monosodium urate or calcium pyrophosphate dihydrate crystals, respectively, in joints and periarticular tissues. These salts and their crystals were shown to activate the NLRP3 inflammasome in vitro and in mouse models, whereas deficiency in NLRP3 and inflammasomal signaling reduced crystal-induced inflammatory activities (Martinon et al., 2006).

Excess cholesterol deposition and formation of cholesterol crystals in artery walls occur early and are a major contributor to the formation of atherosclerotic lesions in arteries to lead to various atherosclerotic cardiovascular diseases (Duewell et al., 2010). Ablation of NLRP3 and other components of the inflammasome reduced atherosclerotic lesions in mouse models. Inhibition of the NLRP3 inflammasome with MCC950 significantly reduced atherosclerotic lesion development in apolipoprotein E-deficient mice (van der Heijden et al., 2017).

In patients with Alzheimer’s disease, amyloid plaques are formed in brain tissue and are associated with the pathogenesis of the disease. Amyloid plaques are composed of oligomers and orderly aggregates or fibrils of the amyloid beta peptide. Extracellular amyloid beta fibrils are engulfed by microglial phagocytes, which clear the fibrils from tissue. Phagocytosis of the fibrils would result in lysosomal damage and release of cathepsin B, leading to activation of the NLRP3 inflammasome and release of proinflammatory and neurotoxic mediators from microglia, which cause inflammatory damage in brain tissue (Halle et al., 2008). The fenamate nonsteroidal anti-inflammatory drugs were shown to inhibit the NLRP3 inflammasome and thereby protected against Alzheimer’s disease in rodent models (Daniels et al., 2016).

Parkinson’s disease is among a group of neurodegenerative disorders known as synucleinopathy that are characterized by the formation of inclusions called Lewy bodies. Lewy bodies are mainly composed of fibrillar *α*-synuclein aggregates. In Parkinson’s disease, Lewy bodies are formed in the dopaminergic neurons in the substantia nigra pars compacta of the brain and is the cause of death of the neurons. Released fibrillar *α*-synuclein and Lewy bodies were shown to be taken up by microglia through phagocytosis, which led to the activation of the NLRP3 inflammasome and secretion of IL-1*β* and other proinflammatory mediators, resulting in strong inflammatory responses in patient brains with Parkinson’s disease (Codolo et al., 2013).

#### 6. Exogenous Particulate-Associated Pathology

Humans encounter exogenous particulates from environmental and workplace exposures as well as medical applications. Exposure to exogenous particulates can lead to detrimental diseases, such as pneumoconiosis and mesothelioma, or, in the case of a medical application, therapeutic effects. Potassium aluminum sulfate is commonly used as an adjuvant in human and animal vaccines and is referred to as alum. The mechanism by which alum stimulates vaccine immune responses is incompletely understood. Alum activated the NLRP3 inflammasome and induced IL-1*β* and IL-18 secretion in vitro, and the in vivo adjuvant effect of alum was dependent on the presence of NLRP3, ASC, and IL-1 (Eisenbarth et al., 2008). In a separate study, alum and crystalline silica, the causal agent for silicosis and lung cancer, were shown to activate the NLRP3 inflammasome by disrupting the lysosome to release cathepsin B, which serves as a sterile DAMP for NLRP3 activation (Hornung et al., 2008). In a separate study, silica and asbestos, the causal agent for asbestosis and mesothelioma, activated the NLRP3 inflammasome in macrophages, which required the efflux of the intracellular potassium and generation of intracellular reactive oxygen species (Cassel et al., 2008). In a rat model of silicosis, silica was shown to activate the NLRP3 inflammasome and release of proinflammatory IL-1*β*, basic fibroblast growth factor, and high mobility group protein B1, resulting in silicosis. Moreover, both the NLRP3 activation and silicosis development were influenced by modulation of the surface properties of the silica particles (Peeters et al., 2014).

#### 7. Cancer and Metastasis

A role of NLRP3 inflammasome-driven inflammation in cancer development was partly supported by the finding that the anti-IL-1*β* therapy using canakinumab in the CANTOS trial is correlated with a reduction in the incidence of lung cancer in the patient population studied (Ridker et al., 2017b). In a mouse study, lack of NLRP3 significantly reduced the tumor burdens of methylcholanthrene-induced sarcomas and from experimental and spontaneous metastasis in a natural killer cell– and IFN-*γ*-dependent manner. These findings indicate that NLRP3 is an important supporter of natural killer cell–mediated control of carcinogenesis and metastasis (Chow et al., 2012). Loss of NLRP3 in mice also increased the survival of mice against melanoma upon vaccination with a dendritic cell vaccine against melanoma cell line B16-F10 by about fourfold relative to control mice. The increased vaccine efficacy in NLRP3-deficient mice reflected differences in myeloid-derived suppressor cells with a fivefold reduction. These and further characterization of the mice supports a role of NLRP3 in impeding antitumor immune responses induced by antitumor vaccination (van Deventer et al., 2010).

## III. Structure of NLRP3 Inflammasome

NLRP3, like all Nod-like receptors, is a member of the signal transduction ATPases with numerous domains (STAND) family of proteins. STAND proteins are typically a part of a regulatory network and exhibit sensing, regulation, and scaffolding activities in a single multidomain protein (Leipe et al., 2004). Activation of NLRP3 and assembly of its inflammasome are largely mediated through protein-protein interactions between evolutionarily conserved, homotypic domains of NLRP3 and other components of the inflammasome. Major efforts were made in the recent years to elucidate the three-dimensional structures of NLRP3 in its inactive state and for its activation and inflammasomal assembly to better understand its function, mode of action, and therapeutic targeting.

### A. Major Components and Domains

**Fig. 2:**
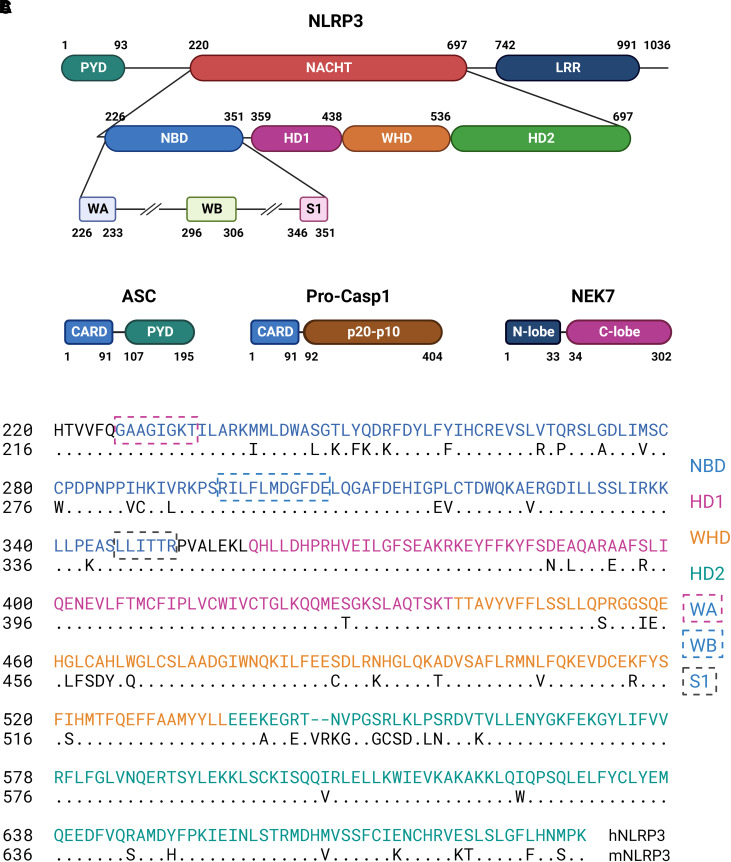
Domain structures of NLRP3 inflammasome proteins. (A) Domains, subdomains, and STAND elements of human NLRP3 as defined for human NLRP3 in UniProtKB/UniProt: Q96P20 and in the atomic structures of PDB: 7PZC (Hochheiser et al., 2022b) and PDB: 7ALV (Dekker et al., 2021), and computational modeling of NLRP3 (Samson et al., 2020). (B) Domains of ASC, Pro-casp1, and NEK7 as defined for human proteins in UniProtKB/UniProt: Q9ULZ3, P29466, and Q9HC98, respectively. (C) Amino acid sequence alignment between human and mouse NACHT domains. Alignment and comparison revealed a percent identify of 86.86% and similarity of 91%. S1, sensor 1; WA, Walker A; WB, Walker B.

The NLRP3 inflammasome is composed of three major protein components: NLRP3, ASC, and Casp1. NLRP3 is a tripartite protein consisting of three distinct domains: an N-terminal PYD domain, a central nucleotide triphosphatase domain known as NACHT characteristic of NACHT STAND proteins, and a C-terminal leucifne-rich repeat (LRR) domain (Fig. 2A). Additionally, a basic region followed by a fish-specific NACHT-associated domain (FISNA) is found between PYD and NACHT, whereas a transition LRR is localized N-terminal to LRR. ASC consists of a PYD and a caspase-recruitment and activation (CARD) domain, while pro-casp1 contains a CARD domain and a p20-p10 domain that is cleaved into p20 and p10 upon activation (Fig. 2B). Activated NLRP3 oligomerizes and recruits ASCs through homotypic binding between their PYDs. The NLRP3-ASC complex in turn recruits multiple pro-casp1s through binding between their CARDs to result in the autocleavage and activation of pro-casp1 and the proteolytic processing and maturation of IL-1*β*. Never in mitosis gene A–related kinase 7 (NEK7) is a mitotic kinase that binds NLRP3 to facilitate its activation at the interphase of cell cycle. NEK7 consists of an N-terminal lobe and a C-terminal kinase lobe (Fig. 2B).

PYD is a conserved protein motif termed death fold found in proteins involved in cell death processes, such as apoptosis and pyroptosis. The PYD domains of NLRP3 and ASC mediate the binding between NLRP3 and ASC as well as among ASCs for speck formation. NACHT domains are found in proteins involved in apoptosis and the transcription of genes encoding major histocompatibility complex class I and class II molecules. NLRP3 NACHT binds ADP/ATP and mediates ATP-induced oligomerization of NLRP3. Structurally, NLRP3 NACHT contains typical STAND elements and shares a high degree of sequence homology between human and rodent proteins (Fig. 2C). LRR domains are evolutionarily conserved domain structures found in proteins associated with innate immunity in plants, invertebrates, and vertebrates. In mammals, LRRs are contained in TLRs and NLRs. Each LRR protein contains 2 to 45 LRRs with each LRR containing 20 to 30 amino acid residues. LRRs generally adopt an arc or hook-like shape. By analogy with the findings on LRRs of several NLRPs with known structures, typified by the NLRC4, the NLRP3 LRR is believed to be involved in ligand sensing and autoregulation of NLRP3 (Hu et al., 2013; Broz and Dixit, 2016). Recent findings revealed that NLRP3 LRRs interact with each other via “face-to-face” and “back-to-back” interfaces and these interactions are the major driving force for the formation of oligomeric ring-like structures of inactive NLRP3 in cells (Andreeva et al., 2021; Hochheiser et al., 2022b; Ohto et al., 2022). The basic region of NLRP3 may mediate its association with negatively charged membrane structures in cells, whereas the FISNA subdomain may facilitate the sensing of ion flux to cause conformational changes necessary for activation (Schroder and Coll, 2021).

### B. Structures of Domains, the Full-Length Protein, and Oligomers of Inactive NLRP3

#### 1. Crystal and NMR Structures of PYD

**Fig. 3:**
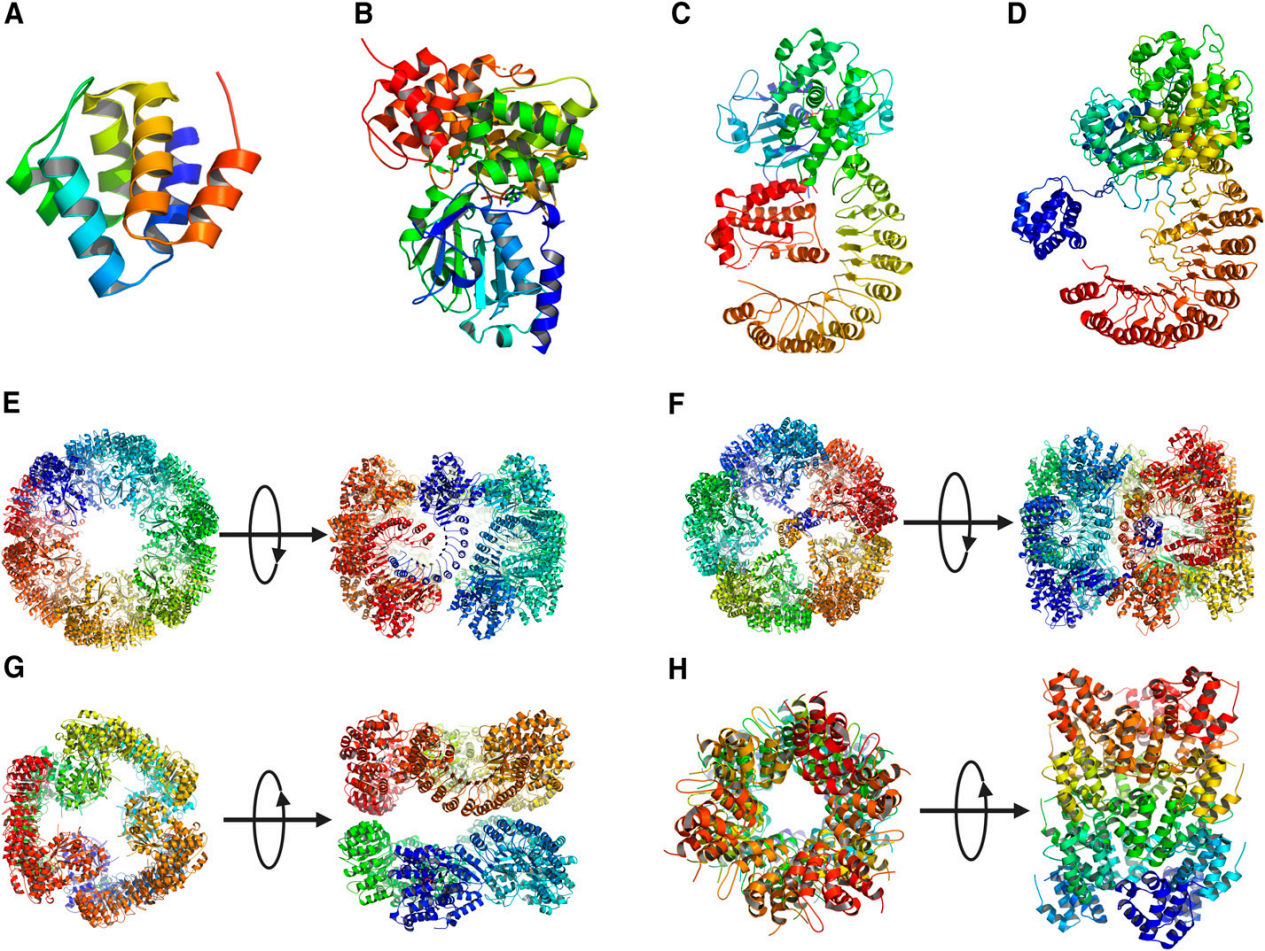
Three-dimensional structures of NLRP3. (A) Crystal structure of human PYD (PDB: 3QF2) (Bae and Park, 2011). (B) Crystal structure of human NACHT (PDB: 7ALV) (Dekker et al., 2021). (C) Cryo-EM structure of human NLRP3 NACHT-LRR bound with NEK7 (PDB: 6NPY) (Sharif et al., 2019). (D) Cryo-EM structure of full-length human NLRP3 derived from the human decamer ring structure (PDB: 7PZC) (Hochheiser et al., 2022b). (E) Double-ring cage of inactive full-length mouse NLRP3 (PDB: 7LFH) (Andreeva et al., 2021). (F) Cryo-EM structure of inactive full-length human NLRP3 (PDB: 7PZC) (Hochheiser et al., 2022b). (G) Cryo-EM structure of inactive human NLRP3 lacking PYD (NLRP3ΔP) (PDB: 7VTP) (Ohto et al., 2022). (H) Human NLRP3 PYD domain filament (PDB: 7PZD) (Hochheiser et al., 2022a). In E–H, both the top-down and side views of the oligomers are shown.

A three-dimensional structure of NLRP3 PYD was obtained by X-ray crystallography at a resolution of 1.7 Å (Fig. 3A) and by solution NMR spectroscopy (Bae and Park, 2011; Oroz et al., 2016). The crystallographic and solution structures of the PYD share a high degree of similarity to each other with a backbone root mean square deviation of 1.66 Å. The overall architecture of NLRP3 PYD exhibits six helices (a1–a6) and five connecting loops, forming the canonical antiparallel six-helical bundle fold characteristic of the death domain family of proteins. This death domain structure is conserved in PYD proteins, but NLRP3 PYD resembles more of those of NLRP4 and NLRP10 than NLRP1, NLRP7, and NLRP12. The antiparallel helical bundles of NLRP3 PYD are held tightly by a central hydrophobic core and further stabilized by a second hydrophobic surface formed by residues Phe32, Ile39-Pro42, Leu57, and Phe61.

NLRP3 PYD plays several roles in inflammasomal assembly. Activated NLRP3 binds ASC via homotypic interactions between their PYDs. Additionally, NLRP3 PYD mediates the oligomerization of NLRP3, whereas ACS PYD mediates prion-like aggregation of ASCs into fibrils. Interactions between PYDs involve both electrostatic and hydrophobic interactions. Binding between NLRP3 and ASC PYDs is mediated through the same binding sites as those involved in the self-assembly of ASC PYDs. The PYD domains have high tendency for aggregation and their binding affinities have been difficult to measure. NMR titration was used to determine the dissociation constant (*K_D_*) values for binding between NLRP3 PYD and ASC PYD and among ASC PYDs, which were estimated to be in the range of 2 to 55 *μ*M (average of 22 *μ*M) and 40 to 100 *μ*M (average of 65 *μ*M), respectively (Oroz et al., 2016). The dynamics and determinants for PYD-PYD binding between NLRP3 and ASC and within ASC fibrils for the formation of the NLRP3 inflammasome are complex. A recent cryo-EM study revealed directional polymerization and extension of ASCs into filamentous structures upon activation of NLRP3 and formation of NLRP3 PYD nucleation leads, which is discussed in more detail in section IV (Hochheiser et al., 2022a). Notably, the crystal structure of NLRP3 PYD revealed a disulfide bond between the Cys8 residue in helix 1 and the Cys108 residue in the loop that connects PYD and nucleotide-binding domain (NBD) in NLRP3 (Bae and Park, 2011). The Cys8 and Cys108 residues of NLRP3 are conserved across species. It was speculated that formation of this disulfide bond upon exposure to ROS contributes to the relieve of autoinhibition that keeps moncomeric NLRP3 in an inactive state during NLRP3 activation by ROS and ROS-stimulating PAMPs and DAMPs.

#### 2. Crystal Structure of NACHT

A recombinant human NLRP3 protein from amino acid residue 131 to 679 (mostly the NACHT domain) was purified and crystalized in the presence of ADP and NP3-146, an MCC950 analog (Dekker et al., 2021). The crystal structure revealed structural features of NACHT and its binding with ADP and NP3-146 at a resolution of 2.8 Å (Fig. 3B). Binding of NP3-146 to NACHT is discussed in more details in a latter section. NLRP3 NACHT consists of four subdomains that are typical of STAND NACHTs and are named the NBD, helical domain (HD) 1, winged helix domain (WHD), and HD2, respectively. The overall subdomain structure of NACHT, including NBD, HD1, WHD, and HD2, is similar to that of the cryo-EM structure of NLRP3 NACHT-LRR in complex with NEK7 discussed in more detail later (Fig. 3C) (Sharif et al., 2019) and to those of NLRC4 and NOD2 (Hu et al., 2013; Maekawa et al., 2016). The crystal structure revealed that the NLRP3 NACHT domain adopts an inactive, closed conformation in the absence of its LRR and NEK7, similarly to the inactive conformation of NLRP3 NACHT-LRR in complex with NEK7.

**Fig. 4:**
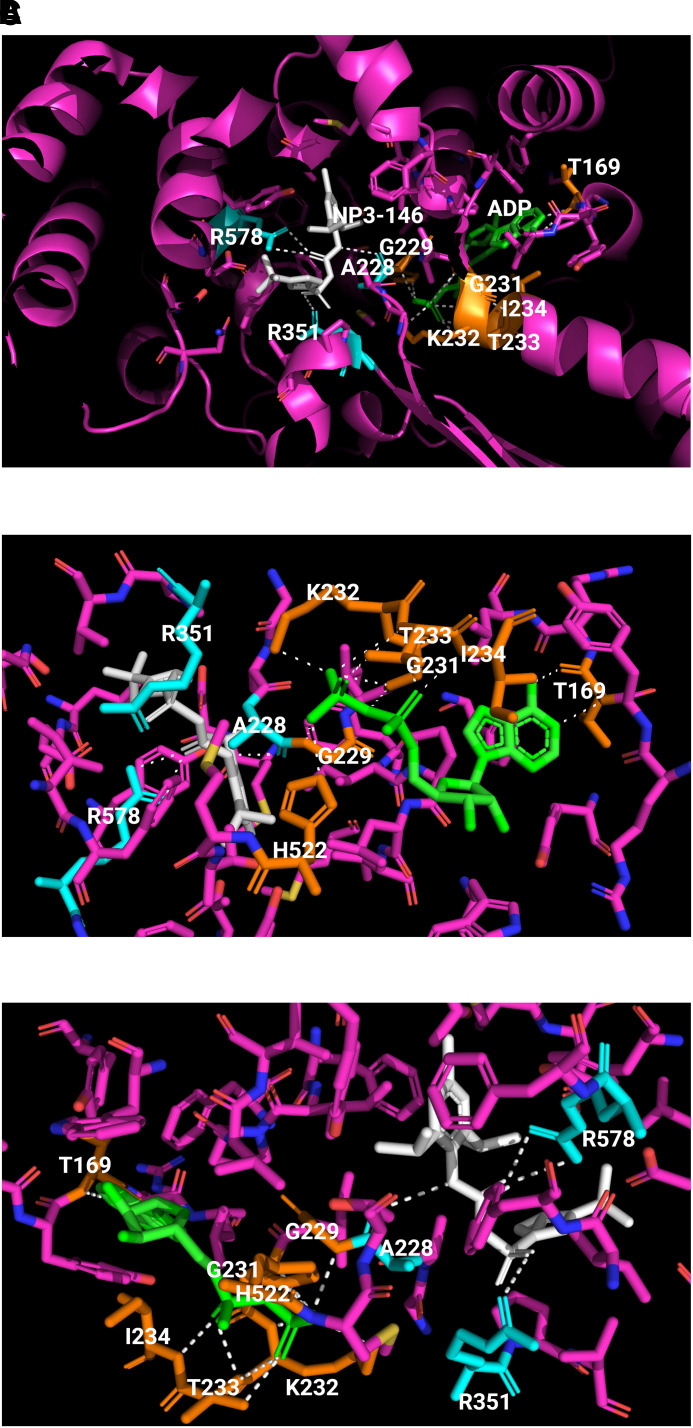
Binding of ADP and an MCC950 analog inhibitor in inactive NLRP3. The crystal structure of human NACHT bound with ADP and MCC950 analog NP3-146 is used to illustrate the binding sites and formation of polar bonds with residues for ADP and NP3-146. In the structure, bound ADP is shown in green, bound NP3-146 in white, residues within 5 Å of a ligand in yellow for the ADP binding site or in cyan for the NP3-146 binding site, and all other residues in magenta. Polarized bonds are shown with dotted line in white. (A) Crystal structure of human NLRP3 NACHT bound with ADP and NP3-146 (PDB: 7ALV) (Dekker et al., 2021). NLRP3 NACHT displays an overall configuration characteristic of NACHT STAND proteins. ADP and NP3-146 bind in adjacent but separate binding pockets. (B) The ADP binding site from A. The *β*-phosphate of ADP forms a polar contact with His522 from the WHD subdomain. ADP makes extensive interactions with the Walker A motif (226-GAAGIGKT-233) including polar bonds with Gly229, Gly231, Lys232, Thr233, and Ile234. Its adenine group forms polar bonds with Thr169. (C) The NP3-146 binding site from A. NP3-146 binds to the opposite side of Walker A from ADP binding. Its urea group forms a crucial polar contact with Ala228 of Walker A. The inhibitor also forms important interactions with Arg578 of HD2, whereas its sulfonyl group interacts with Arg351 of NBD.

The structural motives of NACHT are spatially arranged to enable interactions of key residues to stabilize the inactive conformation and, possibly, to control domain rearrangement upon activation (Fig. 4) (Dekker et al., 2021). The NACHT structure shows typical STAND elements, that is, a Sensor-1 motif (residues 346-LLITTR-351) following a Walker A motif or P-loop (residues 226-GAAGIGKT-233) and a Walker B motif (residues 296-RILFLMDGFDE-306) (Fig. 2A, 2C). ADP binds to the nucleotide-binding pocket of NLRP3 and copurifies as a cofactor. The *β*-phosphate group of ADP interacts with His522 of WHD and has extensive contacts with Walker A, but not Walker B, in agreement with the notion that Walker B plays a role in coordinating the *γ*-phosphate in the presence of ATP and facilitating the hydrolysis of ATP. The interaction between ADP and WHD-His522 likely keeps the NACHT in its closed conformation. The sensor-I motif has a conserved Arg351 on the tip of β4 that may form a salt bridge with Glu527 in the presence of ADP to stabilize the inactive closed conformation. When ATP binds, Arg351 is released from the salt interaction to sense and coordinate the *γ*-phosphate of ATP. The gain-of-function mutation Glu527Lys associated with CAPS supports that loss of the Arg351-Glu527 interaction destabilizes interdomain interactions and thereby disrupts the closed conformation of inactive NLRP3. Arg262 is localized adjacent to Walker B at the tip of the *β*2 strand. Arg262 may form a salt bridge with Glu511, which contributes to the stabilization of the inactive conformation by providing additional interdomain interactions between NBD and WHD. This notion is supported by CAPS mutations Arg262Phe and Arg262Leu, which likely destabilize NBD-WHD interactions to enable autoactivation in CAPS.

#### Cryo-EM Structure of NLRP3 NACHT-LRR in Complex with NEK7

The structure of human NLRP3 with NACHT and LRR domains was resolved by cryo-EM of a recombinant NLRP3 in complex with NEK7 (Fig. 3C) (Sharif et al., 2019). NEK7 binds NLRP3 with a dissociation constant (*K_D_*) of 78.9 ± 38.5 nM (Schmid-Burgk et al., 2016; Shi et al., 2016; Sharif et al., 2019). Binding between NLRP3 and NEK7 is required for NLRP3 activation following potassium efflux in the interphase of cell cycle (He et al., 2016). A complex of a maltose-binding protein-tagged NLRP3 that lacks the PYD domain but is bound with an ADP and an NEK7 was purified and used to obtain the cryo-EM structure of inactive NLRP3 at a resolution of 3.8 Å (Sharif et al., 2019). In this structure, the monomeric NLRP3 NACHT-LRR displays an earring shape with a compact, globular NACHT domain and a curved LRR domain. This overall conformation is also observed in other NLRs, such as NLRC4 (Hu et al., 2013). The structure of NLRP3 NBD-HD1-WHD resembles those of NLRC4 and NOD2, whereas the HD2 and LRR conformations of NLR proteins are generally more variable among NLRs. The NLRP3 LRR consists of 12 repeats with the concave face containing parallel β-sheets and the convex face showing helices and other structures. The LRR encircles the C-terminal lobe of NEK7 as a rigid body. The N-terminal lobe of NEK7 extends away from the complex.

Three interfaces were revealed to mediate NLRP3-NEK7 interactions: those between NLRP3 LRR and the first half of NEK7 C-lobe (interface I) and those between NLRP3 HD2 or NBD and the second half of NEK7 C-lobe (Interfaces II and III, respectively). The interaction between NLRP3 and NEK7 C-lobe is dominated in part by electrostatic complementarity, with the former being negatively charged and the latter being positively charged at a physiologic pH value. Mutational studies of key residues in the interfaces, such as residues Gln129, Arg131, and Arg136 of NEK7 at interface I, residues Ser260, Asp261, and Glu265 of NEK7 at interface II, and Asp290 and Arg294 of NEK7 at interface III, support the importance of interfaces I and II in NLRP3-NEK7 binding (Sharif et al., 2019). Mutational studies also revealed that NEK7 mutants were compromised for supporting NLRP3 activation induced by nigericin in NEK7-knockout immortalized mouse bone marrow–derived macrophages, which supports the role of these residues in mediating NEK7-NLRP3 interaction and is consistent with the function of NEK7 to license NLRP3 for activation. Additionally, the structural study uncovered that binding of NEK7 to NLRP3 prevents NEK7 from binding to NEK9 in mitosis, and vice versa. Therefore, the functions of NEK7 to promote mitosis in the mitotic phase and to license NLRP3 activation in the interphase are mutually exclusive.

#### 4. Cryo-EM Oligomeric Ring-Like Structures of Inactive NLRP3

Three recent studies on the structures of inactive NLRP3 with ADP bound and with or without MCC950 revealed that inactive NLRP3 exists in an equilibrium between monomeric and oligomeric proteins in cells. Oligomeric NLRP3 forms stable ring-like structures that can be enriched and analyzed by cryo-EM (Fig. 3E–3G) (Andreeva et al., 2021; Hochheiser et al., 2022b; Ohto et al., 2022). These oligomers were localized at or were capable of associating with membrane structures in cells, such as the Golgi apparatus and the dispersed *trans*-Golgi network (dTGN). In these structures, the ring-like structures differ from one another considerably regarding the number of monomers and configurations of rings, but the driving force for the formation of all rings appears to be primarily LRR-LRR interactions in all cases. Formation of the ring-like structures is likely to be influenced by the proteins studied and the nucleotides and inhibitors used to stabilize the proteins for purification and cryo-EM study.

Cryo-EM structure determination of mouse full-length NLRP3 expressed and purified from HEK293T cells revealed double-ring cage structures of 12, 14, and 16 monomers (Andreeva et al., 2021). The ADP- and MCC950- bound proteins formed six-, seven-, and eightfold oligomers with 12, 14, and 16 monomers, whereas the NLRP3 purified with dATP bound presented predominantly as a sixfold double-ring cage of 12 monomers at a 4.2 Å resolution (Fig. 3E). The cage is formed by interactions between curved LRRs in face-to-face and back-to-back interfaces. The NACHT domains are barely in contact with each other and assume an inactivate conformation analogous to the NLRP3-NEK7 structure. The PYD domain is shielded within two NACHT-LRR rings. The large face-to-face interface is formed by charge complementarity, whereas the smaller back-to-back interface is less charged and involves hydrophobic interactions. Additionally, the face-to-face interface overlaps with the NEK7-binding surface and is thus not compatible with NEK7-binding. Characterization of NLRP3 and the cage structures revealed that nascent NLRP3 is associated with membrane structures and is in equilibrium with cytoplasmic monomeric NLRP3. This membrane association promotes the double-ring cage formation.

In a separate study, the cryo-EM structure of full-length mouse NLRP3 (residues 1 to 1033) bound with ADP and MCC950 was obtained as a dodecamer ring of 12 monomers at 3.6 Å resolution (Ohto et al., 2022). The dodecamer displayed a barrel-shaped oligomer with a hollow core. Each of the top and bottom sides was formed by six NACHT domains, while the lateral side was formed by 12 LRR domains, with a diameter of 220 Å and a height of 150 Å. Like the cage structure discussed above, the dodecamer was formed via face-to-face and back-to-back interfaces of LRR-LRR interactions, whereas the NACHT domains did not show contact with each other.

The cryo-EM structure of full-length human NLRP3 (residues 3 to 1036) was obtained using a baculovirus expressed, MBP tagged fusion protein (Hochheiser et al., 2022b). The structure reflects homogenous particles of a circular shape with a diameter of 20 nm. The three-dimensional structure at 8–11 Å resolution displayed an overall spherical structure with a pentameric assembly at the polar sites and a meander ring along the equator (Fig. 3F). The LRRs are interlocked with their concave sites at the horizontal plane, whereas the NACHTs assemble into two pentamers at the vertical axis. The conformation of full-length human NLRP3 monomer was deduced from this decamer ring structure (Fig. 3D), which is analogous to the structure of the NLRP3-NEK7 complex (compare Fig. 3C and 3D). ADP binds to NACHT in a similar fashion to the crystal structure of human NACHT. Three interfaces were observed. Besides the face-to-face and back-to-back interfaces (interface A and interface C, respective), an interface B complements interface A to stabilize a homodimer of intertwined LRR domains. Interface C mediates the assembly into the decamer ring (Fig. 3F). The overall conformation is considered a pentamer of dimers instead of a dimer of pentamers.

A human NLRP3 protein lacking PYD (NLRP3ΔP, residues 130 to 1,036) was also expressed and purified with ADP and MCC950 bound for structural determination by cryo-EM. The oligomeric form was shown to be a spheric hexamer with a diameter of 160 Å (Fig. 3G) (Ohto et al., 2022). The structure of NLRP3ΔP presented an inactive and closed conformation like the NLRP3-NEK7 structure. Formation of the hexamer involves two interfaces, that is, a head-to-tail interaction between the C terminus of LRR and the NBD and WHD of NACHT and the back-to-back interaction between LRR3 and LRR6 of two protomers. Like other oligomeric structures of NLRP3, this hexamer of NLRP3 NACHT-LRR was incompatible with NEK7 binding.

## IV. Molecular Activation of NLRP3

The recent elucidation of the structures of NLRP3 domains and intact proteins with or without ligands and associated proteins provided significant insights into the mechanism by which NLRP3 is activated to form a multifaceted functional inflammasome.

### A. Priming and Activation of NLRP3

Activation of the NLRP3 inflammasome by many signals involves a two-step process, that is, the priming and activation of NLRP3 (Fig. 1). Accordingly, signals that induce priming or activation are referred to as signal 1 and signal 2, respectively. This two-step model of NLRP3 inflammasome activation has its origin in the study of induction of IL-1*β* (McKee and Coll, 2020). Early findings revealed that, in addition to transcriptional up-regulation of IL-1*β*, the newly synthesized pro-IL-1*β* protein is cleaved by a protease activity into mature IL-1*β* before it becomes competent for secretion and function (Hogquist et al., 1991; Perregaux et al., 1992).

The NLRP3 protein exists in its latent form at a low level in unstimulated cells. During activation, the protein level of NLRP3 is increased and NLRP3 is maintained in an inactive but activation-competent state. This process is known as priming, which bares similarity to the transcriptional upregulation of IL-1*β* in the two-step model of IL-1*β* induction. Many inflammatory stimuli, exemplified by LPS, act as signal 1 to induce NLRP3, Casp1, and Casp1 substrates, such as IL-1*β* and IL-18. Induction occurs at the transcription level through membrane-bound TLRs, or the cytoplasmic NOD2, pathways. Proinflammatory cytokines TNF-*α* and IL-1*β* contribute to the induction by activating their respective receptors. Activation of the receptors leads to increased transcription of the genes through NF-*κ*B and other proinflammatory transcription factors (Bauernfeind et al., 2009; Franchi et al., 2009).

Post-transcriptional modifications of the NLRP3 protein, such as ubiquitylation, phosphorylation, and sumoylation, take place in unstimulated cells and during the priming and activation of NLRP3 to modulate NLRP3 activation and function (Han et al., 2015). As an example, the TNF receptor-associated factor 6 is an E3 ubiquitin ligase that mediates nontranscriptional priming of NLRP3 through TLR and IL-1R signaling in an E3 ligase-dependent manner (Xing et al., 2017). Priming also occurs via protein-protein interactions between NLRP3 and its binding partners. NEK7 is a mitotic serine/threonine kinase that binds NLRP3 and licenses NLRP3 for activation at the interphase of cell cycle (He et al., 2016).

Metabolic regulation of NLRP3 is becoming an important subject for many diseases (Hughes and O’Neill, 2018). Proinflammatory inducers stimulate a shift of the cellular fuel metabolism from oxidative phosphorylation to aerobic glycolysis and polarization of macrophages to M1 macrophages. These events create a metabolic microenvironment that facilitates the priming of the NLRP3 inflammasome. Conversely, inhibition of glycolysis reduces the priming and induction of IL-1*β* by LPS (Tannahill et al., 2013). Several metabolites, including free fatty acids, such as palmitate, ketone bodies, such as *β*-hydroxybutyrate, and short-chain fatty acids derived from fermentation of intestinal microbiota, have been shown to up-regulate NLRP inflammasome priming and activation. Nonetheless, it remains a challenging task to dissect the exact role and molecular targets of metabolism in NLRP3 priming and activation in physiology and disease, owing to the complex nature of both metabolic regulation and NLRP3 signaling.

Three models of NLRP3 activation have been proposed, despite that considerable knowledge gaps exist in these models to account for the activation and function of NLRP3 inflammasome under varied physiologic and disease conditions.

#### 1. Canonical Activation of NLRP3

Following priming, activation of NLRP3 occurs upon recognition of an NLRP3 activator, that is, signal 2. Full activation of the NLRP3 inflammasome involves the release of autoinhibition of NLRP3, oligomerization of NACHT, formation of ASC specks, recruitment and autoactivation of Casp1, and cleavage and maturation of IL-1*β* and other Casp1 substrates. This multistep activation of the NLRP3 inflammasome leading to Casp1 activation is often termed the canonical pathway of NLRP3 activation (Fig. 1). Because NLRP3 is activated by a variety of activation signals ranging from microbial PAMPs to sterile DAMPs and environmental particles and crystals, elucidation of the mechanism by which NLRP3 activating signals are recognized by primed cells has been a subject of considerable debate and remains to be a challenging task. Several cellular and molecular events have been recognized as critical steps in the canonical activation of NLRP3 by prototypical activators. It is worth noting that these events are not mutually exclusive but may occur in parallel or in sequence to activate NLRP3 in a concerted and activator- and context-dependent manner. However, there remains no single-consensus model for NLRP3 inflammasome activation by all activators of NLRP3 to this date.

##### a. Flux of ions

Efflux of intracellular K^+^ was among the first event to be identified as a key step of NLRP3 activation by many activators with few exceptions (Swanson et al., 2019). These activators include bacterial toxins, extracellular ATP, and various particulates (Muñoz-Planillo et al., 2013). In support of this mechanism, a reduction in the intracellular K^+^ concentration was shown to be sufficient to induce, whereas an increase in the extracellular K^+^ concentration blocked, the activation of NRLP3 inflammasome. Both nigericin and gramicidin are microbial toxins as potassium ionophores. Nigericin induced IL-1*β* maturation through pannexin-1-dependent K^+^ efflux, whereas gramicidin formed pores in lipid bilayers to collapse the transmembrane gradients of Na^+^ and K^+^ (Perregaux and Gabel, 1994; Muñoz-Planillo et al., 2013). Extracellular ATP was shown to activate P2X purinoceptor 7 (P2X_7_) to promote the influx of Ca^2+^ and Na^+^ and to coordinate with TWIK2 to result in K^+^ efflux, leading to NLRP3 activation (Di et al., 2018). Besides inducing priming, LPS activate the complement system and the resulting C3a enhances NLRP3 activation by causing the release of intracellular ATP (Asgari et al., 2013). Particulates, such as alum, silica, and calcium pyrophosphate dihydrate crystals, were shown to induce K^+^ efflux, which was critical for the activation of NLRP3 by these particulates (Muñoz-Planillo et al., 2013).

Activation of NLRP3 by nigericin, alum, silica, urate crystals, and the complement membrane attack complex also require the mobilization of Ca^2+^ (Murakami et al., 2012; Triantafilou et al., 2013). Mobilization of Ca^2+^ occurs when extracellular Ca^2+^ moves across channels in the plasma membrane and the Ca^2+^ stored in the endoplasmic reticulum is released into the cytoplasm. Both pathways are often activated during NLRP3 activation. Mobilization of Ca^2+^ is linked to K^+^ efflux mechanistically. In the case of NLRP3 activation by ATP, ATP was shown to induce weak influx of Ca^2+^ through its receptor P2X_7_, which increased K^+^ efflux via TWIK2. K^+^ efflux in turn stimulated Ca^2+^ mobilization by opening plasma membrane Ca^2+^ channels and by releasing endoplasmic reticulum Ca^2+^ (Murakami et al., 2012; Yaron et al., 2015).

Besides mobilization of Ca^2+^ and efflux of K^+^, efflux of Cl^–^ was implicated in NLRP3 activation. Cl^-^ channel blockers and elevated levels of extracellular Cl^-^ can inhibit, whereas reduced levels of Cl^-^ can enhance, the activation of NLRP3 (Domingo-Fernández et al., 2017; Tang et al., 2017). Efflux of Cl^-^ is mediated through the chloride intracellular channel proteins that are present in the plasma membrane and the cytosol and were shown to be required for activation of NLRP3 by several activators, such as nigericin (Domingo-Fernández et al., 2017; Tang et al., 2017). Cl^–^ efflux may be downstream of K^+^ efflux and affects ASC polymerization, whereas K^+^ efflux promotes NLRP3 oligomerization (Green et al., 2018). Although flux of ions has been shown to be important for NLRP3 activation in various systems, the molecular steps that link between ion flux and activation of NLRP3 remain largely unclear. Discrepancies in the findings on ion flux and NLRP3 activation have been noted and warrant further investigation.

##### b. Rupture of lysosomes

Particles and crystals formed in tissue or encountered from exogenous sources are cleared by macrophages through phagocytosis. Engulfed microorganisms and other materials are generally stored and digested in lysosomes. However, most particles and crystals are resistant to enzymatic catabolism in the acidic environment of lysosomes. Instead, engulfed particulates accumulate in lysosomes, leading to lysosomal damage and leakage and, eventually, lysosomal rupture, resulting in the release of the particulates and lysosomal components, such as the lysosomal protease cathepsins, into the cytoplasm. Lysosomal rupture has been implicated in NLRP3 activation by a range of particulates, including alum, urate crystals, cholesterol crystals, silica particles, and asbestos fibers (Hornung et al., 2008). Lysosomal rupture induced by soluble lysosomotropic dipeptide Leu-Leu-OMe was sufficient to activate NLRP3 (Hornung et al., 2008). Inhibition of cathepsins by broad-spectrum inhibitors suppressed NLRP3 inflammasome activation by particulates, implicating a role of cathepsins in NLRP3 activation. However, genetic deletion or knockdown of individual cathepsins did not appear to affect the activation of the NLRP3 inflammasome by particulate stimuli, possibly due to redundant activities among cathepsins for activation of NLRP3 (Orlowski et al., 2015). Lysosomal rupture induced by Leu-Leu-OMe and by particulates was accompanied by K^+^ efflux and Ca^2+^ influx, suggesting a linkage and convergence between lysosome leakage and ion flux in the activation of NLRP3 (Katsnelson et al., 2016). The substrates of cathepsins that link to NLRP3 activation remain to be identified.

##### c. Generation of ROS

NLRP3 activators differ in their size, shape, and composition but exhibit a common feature in that they all stimulate the production of ROS, which would cause oxidative stress in cells. ROS consist of superoxide anion radical (O_2_^–•^), hydroxyl radical (^•^OH), peroxyl radical (RO_2_^•^), and alkoxyl radical (RO^•^). Certain nonradicals are considered as ROS as they are easily converted to ROS including hypochlorous acid, ozone, singlet oxygen (^1^O_2_), and hydrogen peroxide (H_2_O_2_). ROS avidly interact with proteins, lipids, and nucleic acids and thereby alter or destroy the structure and function of macromolecules in cells (Ma, 2010). Oxidative stress is generally accepted as a contributing mechanism in the pathogenesis of a wide range of disease conditions, including aging, chronic inflammation, diabetes, neurodegeneration, and cancer, where activation of NLRP3 is commonly observed. Under a physiologic condition, the production and catabolism of ROS are well controlled and balanced through complex enzymatic and nonenzymatic mechanisms involved in redox regulation and homeostasis (Ma, 2013).

NLRP3 activators stimulate the cellular production of ROS via several mechanisms. Engulfing of particles and crystals by macrophages creates a state of “frustrated” phagocytosis as the macrophages engulf but fail to clear digestion-resistant particulate materials from the cell. Frustrated phagocytosis stimulates the production of a burst of O_2_^–•^ by NADPH oxidases (Dostert et al., 2008). O_2_^–•^ is catabolized to H_2_O_2_ by superoxide dismutase and H_2_O_2_ is converted to more reactive ^•^OH through Fenton and Fenton-like reactions in the presence of a transition metal, such as iron, to result in oxidative stress (Ma, 2010; 2013).

The mammalian mitochondria consume about 90% of the oxygen in the body to generate ATPs via oxidative phosphorylation. This process is also the major source of ROS in cells that are produced through the one-electron reduction of O_2_ by electrons leaked from the respiratory chain at complex 1 and complex III (Turrens, 1997; Finkel, 2005). ATP and particulates have been shown to stimulate ROS production that was necessary for activation of NLRP3 by the activators (Cruz et al., 2007; Dostert et al., 2008; Zhou et al., 2011). Imiquimod is a ligand of TLR7 and activates NLRP3 in a TLR7-independent manner. Imiquimod was shown to inhibit mitochondrial complex I to result in a burst of ROS production from mitochondria and, consequently, NLRP3 activation. This activation of NLRP3 was independent of K^+^ efflux and lysosomal rupture but required the production of ROS from mitochondria (Groß et al., 2016).

The direct targets of ROS for NLRP3 activation remain unclear. The thioredoxin interacting protein (TXNIP) is a cytoplasmic protein that promotes oxidative stress by inhibiting the thioredoxin-dependent catabolism and disposition of H_2_O_2_. TXNIP was shown to bind and activate NLRP3 to form an inflammasome in cells exposed to H_2_O_2_, urate crystals, and high glucose in an ROS-dependent manner. This finding suggests a molecular link between ROS production and NLRP3 activation via TXNIP (Zhou et al., 2010).

A role of oxidative stress is often suggested by inhibition studies using inhibitors of ROS and ROS production. However, this approach is considered as being indicative but not conclusive for establishing a causative relation between ROS production and a given biologic effect, because ROS inhibitors, such as antioxidants, often have a range of off-target effects that may cause or contribute to the inhibitory effect observed. Therefore, the role of ROS in NLRP3 activation by an activator requires confirmation with approaches other than ROS inhibitors like antioxidants.

##### d. Release of mitochondrial DNA

The mitochondria are thought to have evolved to become an organelle in eukaryote cells from saprophytic bacteria. The circular mitochondrial DNA contains CpG DNA repeats and codes for formylated peptides, which are properties of bacterial DNA. Mitochondrial DNA can be released from cells into the circulation upon injury and may serve as a DAMP for NLRP3 activation (Zhang et al., 2010). After stimulation with various NLRP3 activators, mitochondrial DNA was found to be rapidly released into the cytoplasm and was oxidized (Shimada et al., 2012). Release of mitochondrial DNA into the cytoplasm may involve the opening of the mitochondrial permeability transition pores mediated through ROS and Ca^2+^ (Nakahira et al., 2011). Moreover, oxidized mitochondrial DNA preferentially stimulated NLRP3, whereas nonoxidized DNA activated AIM2 (Shimada et al., 2012). These findings support oxidized mitochondrial RNA as a likely DAMP for activation of NLRP3 upon injury.

##### e. Docking and trafficking with membrane structures

In addition to providing ROS and DNA as DAMPs, mitochondria may serve as a docking site for inflammasome formation. NLRP3 is a cytoplasmic protein associated with the endoplasmic reticulum in unstimulated cells. But it became associated with mitochondria and mitochondria-associated membranes upon activation (Subramanian et al., 2013). Under mitochondrial stress, cardiolipin of the mitochondrial inner membrane is exposed to the outer membrane and binds NLRP3 and Casp1, which was shown to be necessary for inflammasome activation (Iyer et al., 2013). During RNA viral infection and upon stimulation with synthetic RNA polyinosinic-polycytidylic acid, the mitochondrial antiviral signaling protein forms a complex with mitofusin 2, which was shown to activate NLRP3 and direct its translocation to mitochondria (Ichinohe et al., 2013; Park et al., 2013).

Different NLRP3 activators promote the formation of dTGN as a result of disassembly of the *trans*-Golgi network (Chen and Chen, 2018). The phosphatidylinositol-4-phosphate on dTGN can recruit NLRP3 through ionic interactions, thereby serving as a scaffold for NLRP3 aggregation and leading to ASC polymerization and activation of Casp1. Recruitment of NLRP3 to dTGN may be an early and common event that results in NLRP3 inflammasome formation in response to diverse signals. NLRP3 may also translocate to the Golgi apparatus by forming a complex with SREBP2 and the SREBP cleavage-activating protein SCAP (Guo et al., 2018). This ternary complex formation is required for optimal activation of the NLRP3 inflammasome in vivo and in vitro. In this context, the SCAP-SREBP2 serves as a signaling hub integrating cholesterol biosynthesis and inflammasome formation in macrophages during inflammation. A recent structural study revealed double-ring cage structures of oligomers of inactive mouse NLRP3 that are associated with membranes in cells (Andreeva et al., 2021). In particular, the NLRP3 oligomer cages were found to be formed in association with Golgi membranes, which promoted TGN dispersion. dTGN transports NLRP3 cages to the centrosome also named microtubule-organizing center to engage centrosomal NEK7 for NLRP3 activation and inflammasomal speck formation. This study provided a mechanistic link among membrane docking, *trans*-Golgi network dispersion, and activation of NLRP3 via NEK7 associated with the microtubule-organizing center in the nucleus (Andreeva et al., 2021).

#### 2. Noncanonical Activation of NLRP3

Engulfed gram-negative bacteria release LPS from degradation of bacterial walls once inside phagocytes. Cytoplasmic LPS can bind and stimulate Casp11 in mice or Casp4 and Casp5 in humans, resulting in the oligomerization of the caspases and their auto-cleavage and, consequently, the noncanonical activation of the NLRP3 inflammasome (Fig. 1) (Shi et al., 2014). Priming is not necessary as Casp4 is expressed at a high level in human cells. Active Casp4/5/11 cleaves GSDMD to induce pyroptosis through plasma membrane pores formed by the cleaved N-terminal fragment of GSDMD. This process also releases ATP by activating pannexin-1 through Casp11 and induces K^+^ efflux, all which in turn drive the activation and oligomerization of NLRP3, formation of ASC specks, and Casp1-dependent maturation and release of IL-1*β* and IL-18 (Kayagaki et al., 2011; Shi et al., 2014). Oxidized phospholipid 1-palmitoyl-2-arachinonyl-sn-glycero-3-phosphorylcholine (oxPAPC) is an endogenous ligand of Casp11. oxPAPC and LPS bind to Casp11 at different domains to activate Casp11 and trigger NLRP3 inflammasome activation followed by Casp1-dependent IL-1*β* maturation (Zanoni et al., 2016).

#### 3. Alternative Activation of NLRP3

Under certain circumstances, human monocytes stimulated by LPS do not appear to require a second activating signal to activate Casp1-dependent IL-1*β* maturation (Gaidt et al., 2016; Chan and Schroder, 2020). In this process, Casp8, which is generally considered to be an apoptosis initiating caspase in the extrinsic apoptosis pathway, serves as an alternative caspase to cleave IL-1*β* and IL-18, either directly or through the NLRP3 inflammasome (Fig. 1). Activation of alternative NLRP3 inflammasome involves signaling through the TLR4-TRIF-RIPK1-FADD-CASP8 pathway but does not require K^+^ efflux, pyroptosome formation, and pyroptosis induction (Gaidt et al., 2016). Upon prolonged exposure to LPS, murine dendritic cells exhibited increased production and secretion of IL-1*β* via the NLRP3 inflammasome independently of P2X_7_ (He et al., 2013).

In murine macrophages, FADD and Casp8 were found to contribute to NF-*κ*B-dependent priming and post-transcriptional activation of the NLRP3 inflammasome, as loss of FADD or Casp8 in a RIP3-deficient background hampered both the priming and activation of canonical and noncanonical NLRP3 inflammasomes (Gurung et al., 2014). Given that FADD and Casp8 are known mediators of apoptosis, the identification of FADD and Casp8 as upstream regulators of NLRP3 priming and activation suggests cross-interactions between apoptosis and pyroptosis pathways. Indeed, macrophages infected with certain pathogens, such as influenza A virus, vesicular stomatitis virus, *Listeria* monocytogenes, Salmonella enterica serova Typhimurium, or *Yersinia*, or upon TLR priming in the absence of TAK1, exhibited robust inflammatory cell death that are characteristic of pyroptosis, apoptosis, and necrosis, indicating the concomitant activation of all three pathways of cell death, a unique form of inflammatory cell death termed panoptosis (Christgen et al., 2020; Malireddi et al., 2020a,b). Panoptosis is mediated through multifaceted cell death complexes called panoptosomes that likely include NLRP3, ASC, and Casp1 as well as apoptosis and necrosis mediators. Recent studies also identified caspase-6 having a critical role in IAV-induced cell death (Zheng and Kanneganti, 2020). Activated Casp6 participates in the panoptosome assembly and thereby promotes Casp1-mediated cell death in response to IAV infection in alveolar macrophages. Details on the mediators, molecular events, and signaling pathways of panoptosis remain largely unclear and await future investigations.

### B. Structural Insights into NLRP3 Activation and Inflammasomal Assembly

#### 1. Nucleotide Binding and Exchange in NLRP3 Activation

The crystal structure of inactive NLRP3 NACHT has implications for its nucleotide exchange and activation. It is generally believed that activation of STAND proteins involves an ADP/ATP switch where the monomeric ADP-bound state of inactive protein converts to an active, multimeric state with ADP being replaced by ATP (Sandall et al., 2020). NLRP3 exhibits an ATPase activity upon activation but what role the hydrolysis of ATP plays in the activation and function of NLRP3 remains uncertain. ATP binding may facilitate activation by stabilizing the active conformation. Alternatively, ATP hydrolysis may modulate an off-switch mechanism.

Only a low level of ATP binds to purified NLRP3 even when ATP is included in all purification steps. ADP is often used to stabilize inactive NLRP3 in structural studies of NLRP3 and its oligomers (Sharif et al., 2019; Dekker et al., 2021; Hochheiser et al., 2022b; Ohto et al., 2022). Alternatively, dATP can be used to facilitate the purification of full-length mouse NLRP3 and obtain a cage structure of the inactive inflammasome at 4.2 Å resolution (Andreeva et al., 2021). In this study, dATP was shown to be hydrolyzed by NLRP3. Pharmacological inhibition of NLRP3 revealed that certain small chemical inhibitors of NLRP3, exemplified by MCC950 and C77, interfere with ADP/ATP binding and inhibit the ATPase activity of NLRP3, which is discussed in more detail in later sections (Coll et al., 2019; Tapia-Abellán et al., 2019; Sebastian-Valverde et al., 2021). It is also known that ribonucleotide triphosphates, including ATP, GTP, CTP, TTP, and UTP, support Casp1 activation by NLRP1 at nanomolar concentrations in a reconstituted system, whereas nonhydrolyzable ATPs, such as ATP-*γ*-S and GTP-*γ*-S, fail to stimulate NLRP1-dependent Casp1 activation, suggesting a critical role of NTP hydrolysis in NLRP1 activation (Faustin et al., 2007). By analogy with this finding, it is rational to posit that nonhydrolyzable NTPs can be used to clarify the role of ATP hydrolysis in the activation of NLRP3 inflammasome in future studies.

Based on the structure of inactive NACHT and the subdomain rearrangements observed for the activation of NLRC4, a model of active state NLRP3 was proposed (Dekker et al., 2021). This active state NLRP3 displays an elongated molecule as predicted. The NACHT binds ADP but with a reduced affinity, as His522 rotates away from the *β*-phosphate of ADP. The NACHT also presents an accessible nucleotide-binding site, which would facilitate ADP-ATP exchange and support the notion that ATP binding stabilizes the active state of NLRP3.

#### 2. Rotation of NACHT and Oligomerization of NLRP3 into Disc-Like Platform

In view of the structural similarity between NLRP3 and NLRC4, the structure of NLRC4 oligomer was used as a template to generate a computational model for the oligomerization of the NLRP3-NEK7 complex (Sharif et al., 2019). Like NLRC4, this hypothetical NLRP3-NEK7 inflammasome forms a disc-like oligomeric structure that recruits ASCs via homotypic PYD-PYD interactions between NLRP3 and ASC, and for ASC fibril formation through ASC PYDs. In the NLRC4 structure, the NACHT domain undergoes a rigid-body rotation at the HD1-to-WHD junction, leading to the opening of a structure for oligomerization and activation of NLRC4. Similarly, inactive NLRP3 is in a conformation that is not compatible with the oligomeric ring. The hypothetical NLRP3 structure in an active conformation has an approximately 90° rotation of the NBD-HD1 module. This rotation enables the NBD-HD1-WHD module of NLRP3 to directly interact in the ring structure of active NLRP3 without steric hindrance. Additionally, the NLRC4 ring has an LRR-LRR interface between adjacent NLRC4s, whereas the LRR in the NLRP3 oligomer is too short to reach adjacent LRRs, creating gaps in the ring structure. These gaps are filled by NEK7 in the hypothetical NLRP3 inflammasome oligomer ring. Collectively, the study on the NLRP3-NEK7 complex reveals that there are at least two separable steps for the activation of NLR3: (a) binding between NLRP3 and NEK7, which is promoted by priming and recognition of an NLRP3 activator, and (b) conversion of NLRP3 NACHT from an inactive conformation to an active form, which may require ATP binding, ATP hydrolysis, and other allosteric steps (Sharif et al., 2019).

#### 3. Ring-Like Structures and Their Association With Membranes and Membrane Trafficking

The discovery of several oligomeric ring-like structures of inactive NLRP3 and their association with subcellular membranes provided new insights into the mechanisms of autoinhibition of nascent NLRP3, the sensing and activation of NLRP3 by activating signals, and formation of active NLRP3 inflammasome. Under a basal condition, NLRP3 exists in an equilibration between monomeric and oligomeric forms and is localized between the cytosol and subcellular membrane structures. Formation of oligomeric ring structures associated with membranes is a common theme for human and mouse NLRP3s. In these structures, nascent NLRP3 is kept inactive via several mechanisms. These include an inactive configuration of NACHT, buried PYDs that are inaccessible for nucleation of NLRP3 and recruitment of ASC, intertwining LRRs where the binding surface for NEK7 is blocked, and the association of cages with membrane structures that are remote to NEK7 associated with microtubule-organizing center in the nucleus. Formation of NLRP3 oligomer rings or cages at the Golgi apparatus promotes the dispersion of TGNs, which is required for activation of NLRP3 upon treatment with nigericin. The dTGNs may transport the NLRP3 oligomers from the cytoplasm into the nucleus to reach NEK7 for binding to activate NLRP3 at the interface of cell cycle. The basic region following PYD in NLRP3 is likely to mediate membrane association, whereas the FISNA domain may sense ionic flux during activator-induced activation of NLRP3 (Schroder and Coll, 2021).

#### 4. Toward Inflammasomal Assembly: PYD-PYD Interaction and ASC Speck Formation

Formation of the oligomer ring of activated NLRP3 provides a platform for recruiting ASCs through PYD interactions between NLRP3 and ASC PYD and for ASC polymerization into prion-like filament bundles through interactions among ASC PYDs (Figs. 1 and 2). ASC filaments aggregate into speck-like structures observable under microscope. Polymerization of ASCs enables the recruitment of multiple pro-casp1s onto the inflammasome through homotypic CARD-CARD interactions between ASC and pro-casp1, providing a signal amplification hub for inflammasome assembly (Dick et al., 2016). Recruited pro-casp1s are self-activated through proximity-induced protease cleavage to form active Casp1s. Active Casp1 cleaves pro-IL-1*β* and other substrates for their maturation and secretion. Notably, activation of NLRP3 and the subsequent formation of the NLRP3 inflammasome are complex processes regulated by many factors, including priming, post-translational modifications, and allosteric mechanisms. For details on allosteric regulatory mechanisms, such as phosphorylation/dephosphorylation, ubiquitylation/deubiquitylation, and sumoylation, readers are referred to specific and detailed reviews available in the literature (Kelley et al., 2019; Swanson et al., 2019; Chan and Schroder, 2020; McKee and Coll, 2020).

NLRP3 and ASC PYDs are composed of six-helical motives with a characteristic death domain fold (Liepinsh et al., 2003; Lu et al., 2014). The PYDs have a bipolar distribution of its electrostatic surface. While NLRP3 PYDs contribute to the formation of the disc-like structure of NLRP3 oligomer upon activation, ASC PYDs assemble through their oppositely charged surfaces in a back-to-back fashion into ASC filaments. The NACHT domain of NLRP3 or AIM2 was shown to be required for successful ASC polymerization in vitro (Lu et al., 2014), whereas the PYD alone was sufficient to induce ASC speck formation in human embryonic kidney cells (Stutz et al., 2017; Marleaux et al., 2020).

A key step in the process of inflammasome formation is the PYD interaction between NLRP3 and ASC that in turn directs the filamentous extension and speck formation of ASC. To gain insights into this process, the structure of the human NLRP3 PYD filament was resolved by cryo-EM at a 3.6 Å resolution (Fig. 4H) (Hochheiser et al., 2022a). The NLRP3 PYD filament exhibits the same symmetry in rotation and axial rise per subunit as the ASC PYD filament (Lu et al., 2014), with each subunit arranged in a hexagon-like structure, forming three asymmetric interfaces to interact with six adjacent PYDs. At the end of the filaments, complementary interfaces form surfaces named as A- and B-ends. Combined with an in vitro filament reconstitution assay for homotypic transition from NLRP3 PYD nucleation seed to ASC filament elongation, the structural study revealed that ASC filament transition and elongation are unidirectional, originating from the B-end of the NLRP3 filament and toward the formation of prion-like filament bundles and functional inflammasome specks (Hochheiser et al., 2022a).

#### 5. Mutation and Autoactivation of NLRP3

**Fig. 5:**
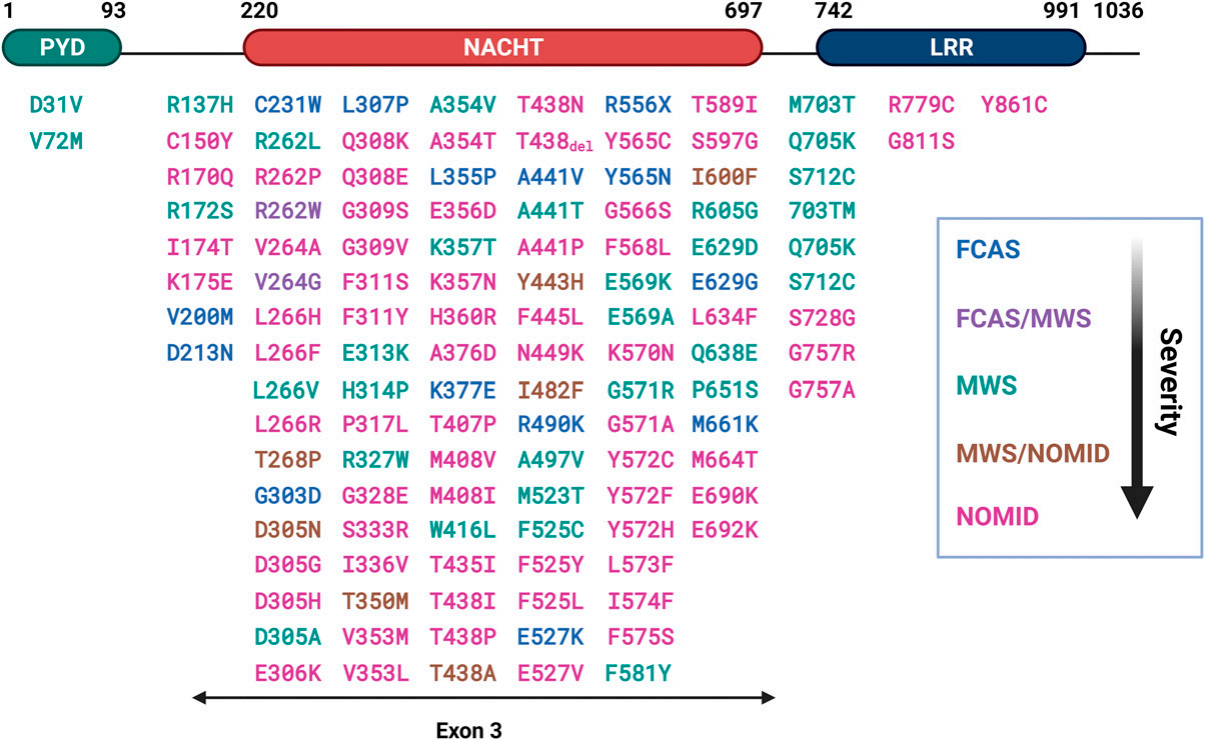
CAPS-associated mutations in human NLRP3. Mutations associated with CAPS were listed in relation to NLRP3 domains and clinical manifestation. Mutations were taken from Infevers (https://infevers.umai-montpellier.fr/) and the literature (Masters et al., 2009; Booshehri and Hoffman, 2019; Samson et al., 2020). Color indicates association with disease types and severity. Mutations were numbered using the first methionine (Met1) as the translation start as defined in the canonical amino acid positions reported in the US National Library of Medicine NCBI and Ensemble, though many mutations were reported using the second methionine (Met3) as the translation start in the literature. Most mutations were found in the NACHT domain encoded by exon 3. Multiple mutations at the same amino acid position indicates a hotspot of mutation.

All mutations associated with CAPS are gain-of-function mutations and are summarized in Fig. 5. About 83% of known mutations of NLRP3 were mapped to the NACHT domain in exon 3. Many of the mutations localize at the NBD-WHD-HD2 interface, which is disrupted during the transition from the inactive to active conformation (Masters et al., 2009; Booshehri and Hoffman, 2019; Samson et al., 2020). It is plausible to postulate that these mutations at the NBD-WHD-HD2 interface lower the energy barrier to enable transition to the active, elongated, and ATP-bound NLRP3 (Tapia-Abellán et al., 2019). This notion is supported by mutations at Arg262 and Glu527 found in CAPS as discussed previously. CAPS mutations may also affect nucleotide binding/hydrolysis, protein stability, or stability of the inactive conformation of NLRP3 (Sharif et al., 2019). In the oligomeric ring structure of inactive human full-length NLRP3, an acidic loop (KEEEEEEKEGRHLD, residues 689-702) in the trLRR domain binds to the concave face of LRR via multiple electrostatic contacts. Y861C is one of the 20 pathogenic CAPS mutations validated at the Infevers database. Y861C is localized at the concave side of LRR that directly interacts with the acidic loop. The remaining 19 mutations concentrate at the interface between NBD and WHD-HD2 in the NACHT domain. Therefore, these pathogenic mutations may disrupt the autoinhibition by altering the configuration of the interfaces that are necessary for autoinhibition.

### C. The Multi-Steps of NLRP3 Inflammasome Activation

The structural findings on NLPR3 and the NLRP3 inflammasome discussed above suggest several major steps in NLRP3 activation. Nascent and inactive NLRP3 exists in cells in an equilibrium between cytoplasmic monomeric and membrane-associated oligomeric forms. Inactive monomeric NLRP3 is bound with ADP in a closed conformation stabilized by interactions among NACHT subdomains and the conserved STAND elements, such as the ADP-His522, Arg351-Glu527, and Arg262-Glu511 interactions (Fig. 4) (Dekker et al., 2021). Sensing of PAMPs and DAMPs through multiple means, including direct interaction, sensing of ionic flux by the basic region, post-translational modifications, and NEK7 binding, may cause domain reorganization sufficient for opening of the nucleotide-binding site to allow ADP/ATP exchange. When His522 is displaced, NLRP3 adopts a fully active conformation. Rotation of NACHT, oligomerization of NLRP3, and ASC speck formation, can occur at multiple steps, depending on whether the involved step is required for the assembly. The oligomeric ring-like and cage-like structures of inactive NLRP3 are largely associated with subcellular membranes like the Golgi apparatus, mitochondria, and lysosomes. Dispersal of TGNs facilitates the transportation of oligomeric NLRP3s into the nucleus for binding with NEK7. Homotypic interactions of NLRP3 PYDs form nucleation leads that direct the unidirectional and prion-like polymerization of ASCs into filamentous structures leading to the formation of inflammasomal specks. Nonetheless, how NLRP3 is activated by diverse PAMPs and DAMPs that have distinct structures and properties under varied physiologic and disease conditions remains largely unclear at molecular levels, awaiting future investigation.

## V. Pharmacological Inhibition of NLRP3 Inflammasome

The NLRP3 inflammasome has become an attractive target for drug development in the recent decade. In particular, it was hoped that inhibition of the NLRP3 inflammasome pathway provides a new means of intervention to stop or demote the progression of chronic disease associated with modern lifestyle, which have become the leading cause of death and for which there remain a lack of effective drug therapy (Mangan et al., 2018; Cross, 2020). Indeed, many drug candidates have been identified through chemical synthesis or by screening natural products and derivatives, some of which have shown potential efficacy in treating diseases in both in vitro and in vivo models, with some reaching clinical trials.

### A. Potential Druggable Targets Relating to NLRP Inflammasome

**Fig. 6:**
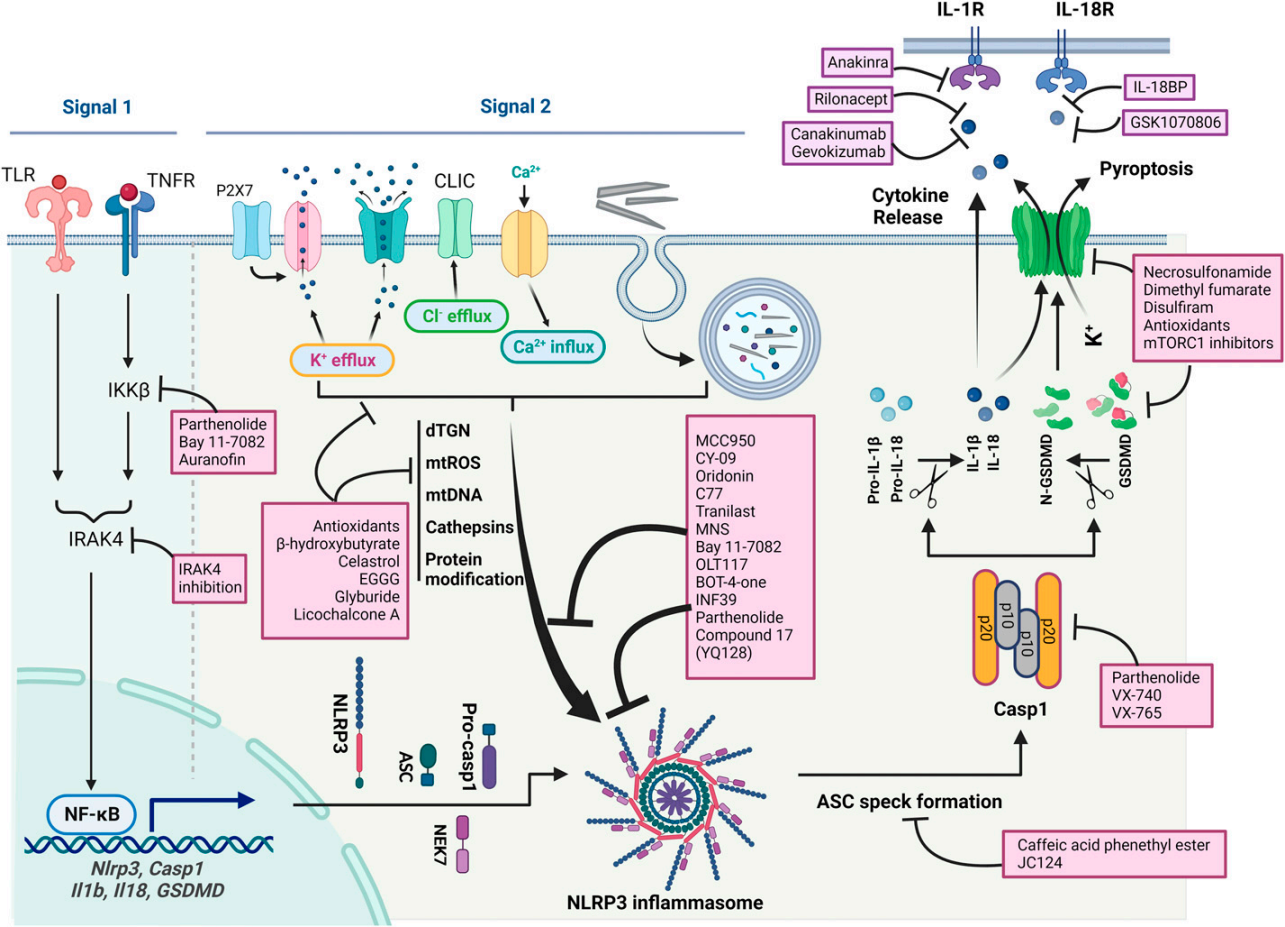
Illustration of inhibition of NLRP3 inflammasome by targeting pathways of activation, signaling, and effectors. Upregulation of the expression of NLRP3 and associated proteins elicited by signal 1 can be targeted for inhibition at key steps of signaling of TLR and TNFR, such as IKK*β* and IRAK4. Activation of NLRP3 by signal 2 involves complex processes and can be inhibited by inhibition of ion flux, ROS production, mitochondrial damage, dTGN, cathepsin release and activity, and various protein modifications. Inhibition of IL1*β* and IL18 and their receptors with neutralizing antibodies, decoy receptors, and natural inhibitors of the receptors have proven to be effective in treating CAPS and several other NLRP3 inflammasome-associated diseases. Inhibition of the NLRP3 inflammasome for its activation and function is perhaps a more direct approach with potentially improved efficacy and reduced side effects and, thus, has raised considerable interest in research and drug development.

From the mechanistic point of view, most steps in NLRP3 inflammasome activation and signaling are potentially druggable targets for pharmacological intervention of NLRP3 inflammasome-associated diseases (Fig. 6).

Inhibition of the priming and licensing of the NLRP3 inflammasome can be achieved by inhibiting the gene transcription, post-transcriptional modification, and interaction with binding proteins of NLRP3, other inflammasome components, and Casp1 substrates (Mangan et al., 2018). In this connection, TLR4, TNFR, and NF-*κ*B are known to mediate the up-regulation of transcription of the NLRP3, Casp1, and IL-1*β* genes in response to specific inflammatory cues (Signal 1). These signaling pathways can be inhibited at several key steps, such as IKK*β* and IRAK4 (Zahid et al., 2019). The signaling events elicited by signal 2 to activate NLRP3 can be targeted by inhibiting several steps upstream of NLRP3, such as ion flux (i.e., K^+^ efflux, Cl^−^ efflux, and Ca^2+^ influx), P2X_7_ signaling, and mtROS production and regulation. Post-transcriptional modifications of NLRP3 are also targets of inhibition of NLRP3 activation (Swanson et al., 2019). Phosphorylation of the Ser3 residue and dephosphorylation of the Ser198 residue repress the activation of NLRP3 by inhibiting NLRP3 homo-oligomerization and its homotypic interaction with ASC PYD (Stutz et al., 2017). Ubiquitylation promotes NLRP3 degradation through the 26S proteasome pathway, whereas deubiquitylation enables its homo-oligomerization (Han et al., 2015; Xing et al., 2017). NLRP3 binds NEK7 at the interphase of the cell cycle, which is required for maintaining NLRP3 in an activation-competent state for activation (He et al., 2016). Inhibition of the interaction between NLRP3 and NEK7 suppresses NLRP3 activation. While all these processes are potential targets for inhibition of NLRP3, most of them are likely to involve cellular processes other than the NLRP3 inflammasome and thus can cause significant off-target effects if inhibited. For example, inhibition of NF-*κ*B represses the induction of NLRP3 protein by LPS but would also suppress many other NF-*κ*B-dependent processes important for innate and adaptive immune functions. It is also uncertain whether inhibition of one target would result in a large reduction in NLRP3 activation, because there are likely alternative and redundant pathways in the priming and licensing of NLRP3.

Inhibition of the effector molecules and their signaling of the NLRP3 inflammasome has proven to be effective in treating CAPS and some NLRP3 inflammasome-driven chronic diseases. These include the U.S. Food and Drug Administration (FDA) approved drugs anakinra, a modified IL-1 receptor antagonist; canakinumab, an IL-1*β* neutralizing antibody; and rilonacept, a dimeric fusion protein consisting of ligand-binding domains of human IL-1R1 and IL-1 receptor accessory protein linked to the human IgG1 Fc fragment to function as a decoy receptor to bind and neutralize IL-1*β* and IL-1*α* (Calabrese, 2002; Dinarello et al., 2012; Jesus and Goldbach-Mansky, 2014). Besides CAPS, anakinra has been used to treat rheumatoid arthritis after treatment with other disease-modifying antirheumatic drugs has failed. Anakinra was also recently approved for treating some COVID-19 patients with pneumonia in Europe. The CANTOS study showed that canakinumab reduced the incidence of atherosclerotic diseases in patients with high C-reactive protein levels (Ridker et al., 2017a). Canakinumab also reduced the incidence of arthritis and gout. Rilonacept was the first drug approved by the FDA to treat recurrent pericarditis besides its use for CAPS. The clinical efficacies of these drugs strongly support the NLRP3 inflammasome substrates and their signaling as a promising target for treating inflammatory diseases. However, these protein-based inhibitors each carry limitations. For instance, anakinra has a plasma half-life of 4 to 6 hours and thus requires daily injections, causing pain and other adverse effects at the site of injection. Protein-based drugs can be expensive, creating substantial economic burden on patients. Blockade of IL-1 may increase the risk for infection, as IL-1-mediated responses are important for defense against many pathogens. For instance, in the CANTOS trial, anti-inflammatory therapy with canakinumab at a dose of 150 mg every 3 months significantly reduced the rate of recurrent cardiovascular events than placebo, independently of lipid-level lowering; but the therapy was associated with a higher incidence of fatal infection than was placebo (Ridker et al., 2017a). Lastly, inhibition of IL-1*β* may not be sufficient for treatment of certain disease phenotypes if the underlying pathology is caused by NLRP3 inflammasome effect molecules other than IL-1*β*. In this regard, IL-18 and GSDMD have been shown to contribute to disease progression in various disease models independently of IL-1*β*.

**Fig. 7:**
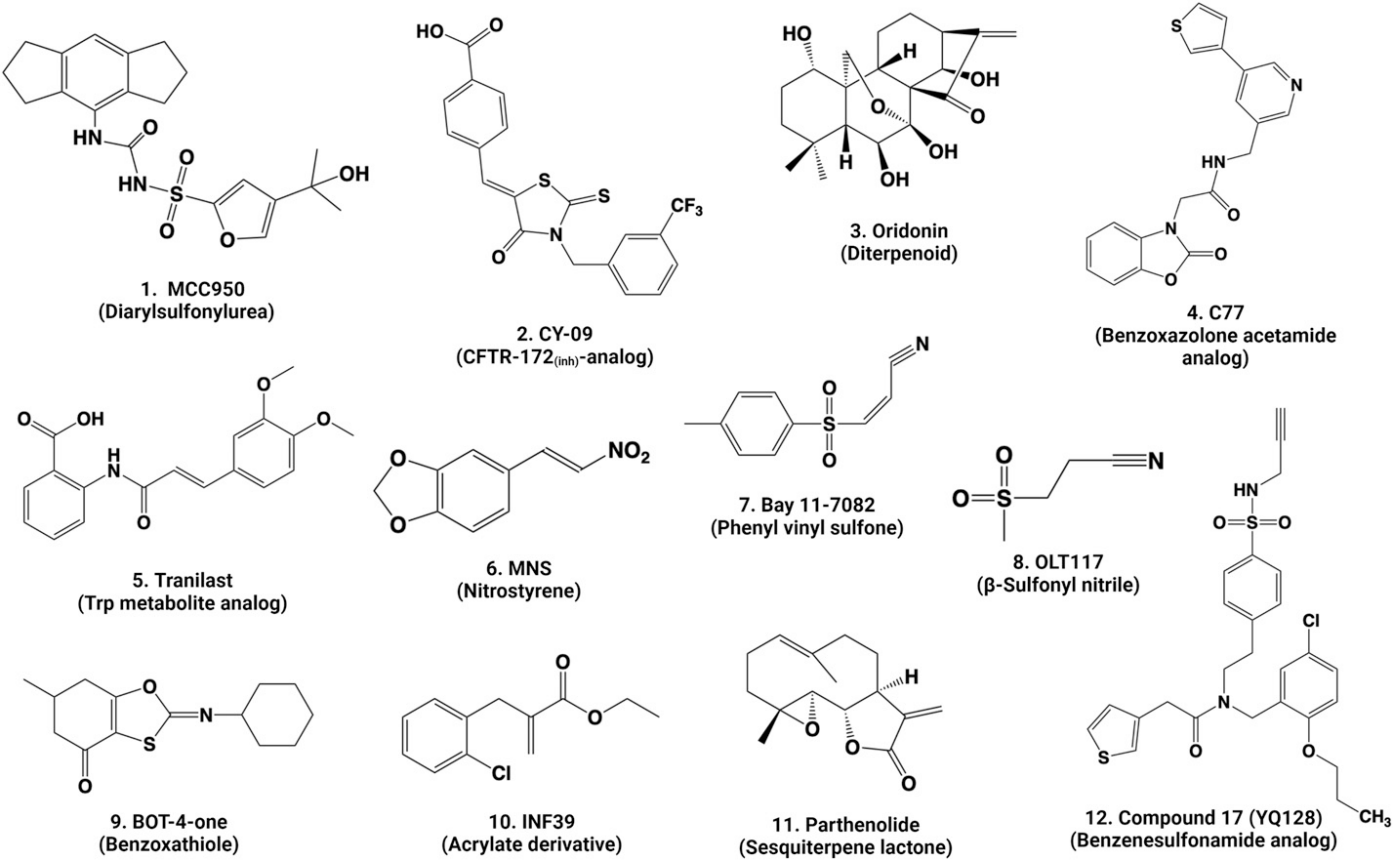
Structures of inhibitors of NLRP3 and its inflammasome. Structures are shown as listed in Table 2 and discussed in the text.

Targeting the NLRP3 inflammasome components provides a seemingly more direct approach to inhibition of the NLRP3 inflammasome. Inhibition of ASC and Casp1 in their agglomeration, activation, and signaling can effectively suppress the formation and function of the NLRP3 inflammasome. Inhibition of the NLRP3 protein itself by small molecule inhibitors is perhaps by far the most direct and rational strategy to block or repress NLPR3 inflammasome-driven inflammation. Compared with protein-based biologicals, small chemical inhibitors of NLRP3 offer several advantages. Small chemical-based inhibitors are generally cost-effective and can be administered orally and thus are less invasive. They likely provide a better specificity for NLRP3 inflammasome-driven processes with reduced off-target effects and more favorable pharmacokinetics. As such, a list of NLRP3 inhibitors have been identified and are actively pursued for therapeutic development (Table 2, Fig. 7).

**TABLE 2:** Inhibitors of NLRP3 and mechanisms of inhibition

Agent	Target (IC_50_)*^a^*	Mechanism of Inhibition	Disease Model and Clinical Trial	Ref*^b^*
MCC950 (CRID3, CP-456773)	NLRP3 (7.5 nM, BMDM) (8.1 nM, HMDM)	Binding to NLRP3 NACHT. Inhibition of ATP/ADP exchange and ATP hydrolysis. Blocking of canonical, noncanonical, and alternative activation of NLRP3	Mouse models of EAE, CAPS, peritonitis, etc. Phase II trial	1
CY-09	NLRP3 (5 *μ*M, BMDM)	Binding to Walker A motif of NLRP3 NACHT to inhibit ATPase and oligomerization. Analog of CFTR_(inh)_-172 without inhibitory activity on CFTR	Mouse models of CAPS and type 2 diabetes. Ex vivo gout patient monocytes	2
Oridonin	NLRP3 (0.5 *μ*M, BMDM)	Covalent binding to NLRP3 NACHT Cys273 to block NLPR3-NEK7 interaction	Mouse models of peritonitis, gouty arthritis, and type 2 diabetes. Anti-inflammation herbal medicine	3
C77	Microglial NLRP3 (4.1 *μ*M) NLRC4 (9.15 *μ*M) (Human THP-1)	Binding to NACHT of NLRP3 and NLRC4 to inhibit ATPase and IL-1*β* secretion in microglial cells. Identified through in silica screening and in vitro and in vivo verification	Inhibit IL-1*β* production in brain regions of mice exposed to LPS, including frontal cortex, cortex, hippocampus, and cerebellum	4
Tranilast	NLRP3 (25 *μ*M, BMDM)	Binding to NLRP3 NACHT to block oligomerization	Mouse models of Gouty arthritis, CAPS, and type 2 diabetes. Anti-allergic drug	5
MNS	NLRP3 (2 *μ*M, BMDM) Syk, Src	Binding to NLRP3 NACHT by targeting cysteine residues to inhibit ATPase and oligomerization. Inhibition of Syk and Src kinases	Antiplatelet effect possibly by inhibition of tyrosine kinases	6
Bay 11-7082	NLRP3 (5 *μ*M, BMDM) NLRC4 IKK-*β*, E2/3, & PTP	Inhibition of NLRP3 NACHT ATPase, possibly by alkylating cysteine thiol. Partial inhibition of NLRC4. Inhibition of IKK-*β*, E2/3, and PTP	Broad anti-inflammatory activity	7
OLT117	NLRP3 (1 nM, mouse J774A.1 macrophages; 1 *μ*M, HMDM)	Inhibition of NLRP3 NACHT ATPase and blocking of NLRP3 activation	Mouse LPS- induced systemic inflammation. Phase II trial	8
BOT-4-one	NLRP3 (0.54-1.28 *μ*M, BMDM) NLRC4	Alkylating agent. Inhibition of NLRP3 NACHT ATPase via alkylation. Induction of ubiquitination of NLRP3. Inhibition of IKK-*β*	Mouse MSU-induced peritonitis. Immunomodulation of dermatitis and arthritis	9
INF39	NLRP3 (10 *μ*M, human THP-1)	Covalent biding to NLRP3 NACHT to inhibit ATPase and canonical and non-canonical activation of NLRP3	Rat DNBS-induced colitis	10
Parthenolide	NLRP3 (5 *μ*M, BMDM) NLRP1, caspase 1, IKK-*β*	Alkylating agent. Inhibition of NLRP3 NACHT ATPase, caspase 1, and IKK-*β*	Anti-inflammation herbal medicine	11
Compound 17 (YQ128)	NLRP3 (0.3 *μ*M, mouse J774A.1 macrophages)	Second-generation benzenesulfonamide inhibitor of NLRP3. Selective inhibition of NLRP3 in vitro and in vivo Brain BBB penetrating	LPS induced activation of NLRP3 in peritoneal macrophages in mice. Analog JC-124 ameliorates (a) amyloid pathology in mouse model of AD, (b) neuroinflammation in mouse TBI, and (c) infarct size in mouse AMI	12

*^a^*IC_50_, the concentration of a drug to result in 50% inhibition of a maximal activity. The IC_50_ for inhibition of induced production of IL1*β* in cultured macrophages and monocytes by common inducers, such as LPS, ATP, nigericin, and mineral particles, is often used to describe the potency of an inhibitor to inhibit the NLRP3 inflammasome. The types of cells used to determine IC_50_ are listed under each IC_50_ value.

*^b^*Reference cited: 1. Coll et al., 2019; Coll et al., 2015; Perregaux et al., 2001; Tapia-Abellán et al., 2019; 2. Jiang et al., 2017; 3. He et al., 2018; 4. Sebastian-Valverde et al., 2021; 5. Huang et al., 2018; 6. He et al., 2014; 7. Jiang et al., 2017; Juliana et al., 2010; 8. Marchetti et al., 2018; 9. Shim et al., 2017; 10. Cocco et al., 2017; Pellegrini et al., 2018; 11. Jiang et al., 2017; Juliana et al., 2010; 12. Jiang et al., 2019.

AD, Alzheimer’s disease; AMI, acute myocardial infarction; BBB, blood-brain barrier; BMDM, mouse bone marrow-derived macrophage; CFTR, cystic fibrosis transmembrane conductance regulator; CRID, cytokine release inhibitory drug; DNBS, 2,4-dinitrobenznesulfonic acid; EAE, experimental autoimmune encephalomyelitis; HMDM, human monocyte-derived macrophage; IKK, I*κ*B kinase; LPS, lipopolysaccharide; MNS, 3,4-methylenedioxy-beta-nitrostyrene; MSU, monosodium urate; NACHT, domain present in NAIP, CIITA, HET-E, and TP1; NEK7, never in mitosis gene A–related kinase 7; NLRC, nucleotide-binding domain (NB) and leucine-rich repeat (LRR) containing receptor (NLR) caspase-recruitment and activation domain (CARD)-containing; NLRP3, nucleotide-binding, oligomerization domain (NOD)-like receptor (NLR) family pyrin domain containing 3; PTP, protein tyrosine phosphatase; Src, Src kinase; Syk, spleen tyrosine kinase; TBI, traumatic brain injury; Trp, tryptophan.

### B. Natural Products and Derivatives as Inhibitors of NLRP3 Inflammasome

Natural compounds have been a major source of traditional medicines and for drug discovery in the treatment of inflammation and inflammatory diseases throughout much of the history of human medicine. Although screening synthetic chemical libraries has been a popular approach in pharmaceutical and academic research to identify potential drugs for several decades, the low success rate of screening small chemical libraries has limited its use in drug discovery and has prompted a renewed interest in screening natural compounds for therapeutic development. The NLRP3 inflammasome has become an ideal target for screening natural products as anti-inflammatory drugs, due to its prominent role in disease pathogenesis as well as having available defined molecular and cell-based bioassays and animal models of various diseases. Many natural products have been screened for inhibition of NLRP3 inflammasome-mediated IL-1*β* production in vitro and for treating NLRP3 inflammasome-associated diseases in vivo. About 39 natural compounds have been found to exhibit anti-NLRP3 inflammasome activities and some with apparent efficacies for treating diseases in animal models. Some natural products-based inhibitors, such as oridonin and parthenolide, are discussed later. For detailed discussion on natural compound inhibitors of the NLRP3 inflammasome, readers are referred to specific reviews available in the literature (Tőzsér and Benkő, 2016; Jahan et al., 2017; Liu and Yu, 2021).

### C. Major Drug Leads Targeting NLRP3 Inflammasome

The list of potential drugs targeting the NLRP3 inflammasome has grown rapidly. Table 2 and Fig. 7 summarized major candidates, most of which are inhibitors of NLRP3.

#### 1. MCC950

Phenotypical screening of IL-1*β* production from activated monocytes and macrophages has been the major means of identifying small molecule inhibitors of the NLRP3 inflammasome. MCC950, initially named as CP-456,773 or CRID3, is the best studied NLRP3 inhibitor. Because of its high potency and specificity in inhibiting the NLRP3 inflammasome, it has been used extensively as a prototypical inhibitor and a research tool for investigation of the mechanism of inhibition of NLRP3 and its role in disease pathogenesis.

Early observations that production of IL-1*β* is a two-step process and that modulation of ion flux specifically affects the maturation and secretion of IL-1*β* prompted a screening of ion flux modulating agents for inhibition of IL-1*β* maturation (Perregaux et al., 2001). Glyburide, a sulfonylurea-containing, insulin secretion-stimulating anti-diabetic drug, was found to block IL-1*β* production in a dose-dependent manner with the half maximal inhibitory concentration (IC_50_) at 12 *μ*M in human peripheral blood mononuclear cells. Inhibition appeared to be independent of its activity to inhibit the ATP-activated K^+^ channel—a pharmacological target of glyburide to stimulate insulin secretion in *β* cells, because a closely related sulfonylurea anti-diabetic drug glipizide inhibited the K^+^ channel similarly to glyburide but failed to inhibit ATP-induced IL-1*β* processing at concentrations of ≤100 *μ*M. Further screening of structural analogs of glyburide identified two diarylsulfonylureas with improved potency: CP-424,174 and CP-412,245, with IC_50_ values of 0.21 *μ*M and 0.26 *μ*M, respectively, for blocking ATP-induced IL-1*β* post-translational processing in human monocytes (Perregaux et al., 2001). Inhibition was specific for production of IL-1*β*, as inhibition was not observed for induced production of TNF-*α* and IL-6. Inhibition was also observed for induced production of IL-1*β* in vivo in animal models.

After the NLRP3 inflammasome was identified as a major mediator of IL-1*β* maturation, the CRID compounds were examined for inhibition of the inflammasome. Through this screening, CRID3 (i.e., CP-456,773) was found to effectively inhibit IL-1*β* secretion and Casp1 processing in response to stimulation of NLRP3 (Coll et al., 2011). CRID3 was renamed as MCC950 and was shown subsequently to inhibit the activation of NLRP3 with IC_50_ values for inhibition of IL-1*β* release at 7.5 nM in mouse bone marrow derived macrophages (BMDMs) and 8.1 nM in human monocyte derived macrophages, respectively (Coll et al., 2015). MCC950 inhibited both the canonical and noncanonical activation of NLRP3 by all known activators. Moreover, inhibition was highly specific for the NLRP3 inflammasome, as it did not inhibit the activation of the AIM2, NLRC4, and NLRP1 inflammasomes or TLR pathways. MCC950 blocked NLRP3-induced ASC oligomerization but did not block K^+^ efflux, Ca^2+^ influx, and ligand-independent, direct NLRP3-ASC interactions. MCC950 inhibited NLRP3 in vivo and was efficacious against the hypersensitive NLRP3 mutations associated with MWS. These findings suggest that MCC950 binds to NLRP3 itself or a target closely linked to the activation of NLRP3 (Coll et al., 2015).

MCC950 has been shown to be efficacious in suppressing a broad range of inflammatory diseases in animal models in which the NLRP3 inflammasome is implicated. For example, MCC950 attenuated the severity of experimental autoimmune encephalomyelitis, an animal model of multiple sclerosis and rescued neonatal lethality in a mouse model of CAPS (Coll et al., 2015). MCC950 improved nonalcoholic fatty liver disease pathology and fibrosis in obese diabetic mice (Mridha et al., 2017). MCC950 also improved the lifespan in an animal model of human Hutchinson-Gilford Progeria disease characterized by an aging syndrome by suppressing NLRP3-driven inflammation (González-Dominguez et al., 2021). Given the efficacy of MCC950 in a variety of inflammatory diseases, it was tested in phase II clinical trials for treatment of rheumatoid arthritis but was withdrawn from the trial, as patients taking the drug displayed liver toxicity with elevated serum enzyme levels. To date, MCC950 remains the most potent and specific inhibitor of the NLRP3 inflammasome and is widely used in mechanistic studies for NLRP3 inhibition and evaluation of NLRP3 function in animal models of diseases. MCC950 was shown to inhibit NLRP3 by binding at or close to the Walker regions of the NACHT domain of NLRP3, as discussed in more details in the following section.

#### 2. CY-09

CY-09 is an analog of CFTR_(inh)_-172 (C172), an inhibitor of the cystic fibrosis transmembrane conductance regulator (CFTR) channel, but is devoid of CFTR inhibitory activity. CY-09 inhibited Casp1 activation and IL-1*β* secretion induced by monosodium urate, nigericin, and ATP at a dose range of 1-10 *μ*M in LPS-primed BMDMs (Jiang et al., 2017). CY-09 did not affect LPS-induced production of TNF-*α* and the expression of pro-IL-1*β* and NLRP3. CY-09 was shown to bind the ATP-binding motif of NLRP3 NACHT domain and inhibit the ATPase activity, thereby inhibiting NLRP3 inflammasome assembly and activation. CY-09 showed apparent therapeutic effects on mouse models of CAPS and type 2 diabetes. CY-09 was also active ex vivo on synovial fluid cells collected from patients with gout.

#### 3. Oridonin

Oridonin is a diterpenoid derived from the traditional Chinese medicinal herb Rabdosia rebescens with significant anti-inflammatory activities. Oridonin was found to be a specific and potent inhibitor of the NLRP3 inflammasome with an IC_50_ value of 0.5 *μ*M in BMDMs (He et al., 2018). Oridonin forms a covalent bond with the Cys279 residue of NLRP3 NACHT domain, thereby blocking the interaction between NLRP3 and NEK7 and the assembly and activation of the NLRP3 inflammasome. In animal models, oridonin was shown to have preventive and therapeutic effects against peritonitis, gouty arthritis, and type 2 diabetes.

#### 4. C77

A virtual screening targeting the ATP-binding site of the NLRP3 model led to the identification of potential inhibitors of the NLRP3 inflammasome that exhibited inhibitory activities toward activation of NLRP3 inflammasome for IL-1*β* production in microglial cells (Sebastian-Valverde et al., 2021). Structural analysis revealed that the inhibitors share a potential pharmacophore of benzoxazolone acetamide. This finding led to the identification of several benzoxazolone acetamide analogs, exemplified by C77, a benzoxazolone acetamide attached to a thiophenyl-pyridinyl substitute through a methylene linker, as ATP-binding site targeting inhibitors of NLRP3. The inhibitors also suppressed the activation of NLRC4 but to a lesser extent than for activation of NLRP3. C77 inhibited IL-1*β* production from microglial cells with IC_50_ values of 4.1 *μ*M for NLRP3 and 9.15 *μ*M for NLRC4 in THP-1 human monocyte derived macrophages.

Treatment of mice with C77 inhibited LPS-induced production of the protein, but not mRNA, of IL-1*β* in brain regions, including the cortex, front cortex, hippocampus, and cerebellum, indicating its potential as a CNS-targeting inhibitor of both NLRP3 and NLRC 4 inflammasomes. Both NLRP3 and NLRC4 inflammasomes have been shown to be involved in neurodegenerative diseases, including Alzheimer’s disease, Parkinson’s disease, and multiple sclerosis (Liu and Chan, 2014; Freeman et al., 2017; Soares et al., 2019; Saadi et al., 2020; Sebastian-Valverde et al., 2021). Inhibition of both inflammasomes by C77 in neuronal tissues likely represents a better strategy for treating diseases associated with neurodegeneration. C77 potently inhibited both NLRP3 and NLRC4, but not AIM2, TNF-*α*, or IL-6 pathways, consistent with its being an inhibitor of the inflammasomes by binding to their NACHT domains. This notion was further supported by the observation that C77 inhibited the NLRP3 ATPase activity at the protein level with an IC_50_ of 40 nM (Sebastian-Valverde et al., 2021).

#### 5. Tranilast

Tranilast is a tryptophan metabolite analog and is an anti-allergic drug. Tranilast is a direct inhibitor of NLRP3 by binding to the NACHT domain and it suppresses the inflammasome assembly by blocking the oligomerization of NLRP3 (Huang et al., 2018). Inhibition is in the concentration range of *μ*Ms with an IC_50_ value at 25 *μ*M in mouse BMDMs. Tranilast did not inhibit the NLRC4 or AIM2 inflammasome, showing a high specificity for inhibition of NLRP3 NACHT. Tranilast exhibited preventive and therapeutic effects toward gouty arthritis, CAPS, and type 2 diabetes in animal models (Huang et al., 2018).

#### 6. MNS

3,4-Methylenedioxy-beta-nitrostyrene (MNS) is an inhibitor of protein kinases like Syk and Src and it inhibits platelet aggregation. MNS was found to inhibit NLRP3 activation with an IC_50_ of 2 *μ*M in BMDM cells (He et al., 2014). Inhibition involved blockade of NLRP3-mediated ASC speck formation and aggregation without affecting K^+^ efflux. Inhibition of NLRP3 is independent of Syk inhibition and requires its nitrovinyl group for NLRP3 inhibition (He et al., 2014). MNS may bind to the NACHT domain and the LRR domain and inhibits the ATPase activity of NLRP3. MNS did not inhibit NLRC4 or AIM2.

#### 7. Bay 11-7082

Bay 11-7082 is a phenyl vinyl sulfone with a broad anti-inflammatory activity and is known to inhibit NF-*κ*B and IKK-*β*. Bay 11-7082 and analogs were shown to inhibit the NLRP3 inflammasome activity in BMDM macrophages independently of its inhibitory effect on NF-*κ*B (Juliana et al., 2010). In vitro assays revealed that bay 11-7082 inhibits the ATPase activity of NLRP3, which in part accounts for its inhibitory activity on NLRP3. Inhibition of NLRP3 by bay 11-7082 may involve alkylation of NLRP3 cysteine residues. Bay 11-7082 did not inhibit the NLRP1 inflammasome but had partial inhibitory effect on Salmonella-induced Casp1 activation through the NLRC4 inflammasome.

#### 8. OLT1177

OLT1177 is an orally active *β*-sulfonyl nitrile compound that potently inhibited the NLRP3 inflammasome activation with IC_50_ values of 1 nM in mouse macrophages (J774A.1) and 1 *μ*M in human macrophages (human monocyte derived macrophages) (Marchetti et al., 2018). OLT1177 reduced the release of IL-1*β* and IL-18 following canonical and noncanonical NLRP3 inflammasome activation, without affecting the activation of the NLRC4 and AIM2 inflammasomes. OLT1177 may directly interact with NLRP3 as it inhibited the ATPase activity of NLRP3 and prevented NLRP3-ASC and NLRP3-Casp1 interactions. In mice, OLT1177 inhibited LPS-induced systemic inflammation. The compound is in a phase II clinic trial.

#### 9. BOT-4-One

BOT-4-one is a benzoxathiole derivative that inhibited NLRP3 activation with an IC_50_ value in the range of 0.54 to 1.28 *μ*M in mouse BMDM cells. BOT-4-one binds to NLRP3 NACHT by alkylating the protein, thereby impairing its ATPase activity (Shim et al., 2017). BOT-4-one also increased the level of ubiquitination of NLRP3, which may contribute to its inhibitory activity on NLRP3. In vivo, BOT-4-one was shown to be effective in suppressing monosodium urate–induced peritonitis in mice and have activities on immunomodulation of dermatitis and arthritis.

#### 10. INF39

INF39 is an acrylate derivative identified as an inhibitor of NLRP3 for treatment of irritable bowel disease (Cocco et al., 2017). INF39 inhibited the canonical and noncanonical activation of NLRP3 with an IC_50_ of 10 *μ*M in human THP-1 derived macrophages. INF39 was shown to be a nontoxic, irreversible inhibitor of NLRP3 by binding to NLRP3 NACHT domain to decrease IL-1*β* release from macrophages. In vivo studies revealed that INF39 alleviated the effects of colitis elicited by 2,4-dinitrobenzenesulfonic acid in rats following an oral administration of INF39.

#### 11. Parthenolide

Parthenolide is a sesquiterpene lactone alkylating agent derived from a medicinal herb. Parthenolide inhibits NF-*κ*B, but its inhibitory activity toward NLRP3 inflammasome was found to be independent of NF-*κ*B inhibition (Juliana et al., 2010). Pathenolide inhibited NLRP3 activation with an IC_50_ of 5 *μ*M in BMDM macrophages. The compound alkylated NLRP3 NACHT and inhibited the ATPase activity of NACHT. Besides NLRP3, pathenolide also inhibited NLRP1, NLRC4, IKK-*β*, and Casp1. Therefore, parthenolide may present off-target effects when used as an NLRP3 inhibitor.

#### 12. Compound 17

Compound 17 (YQ128) is a second-generation NLRP3 inhibitor derived from benzenesulfonamides (Jiang et al., 2019). The initial drug lead, JC124, was shown to inhibit the NLRP3 inflammasome with an IC_50_ value of 3.25 *μ*M in mouse J774A.1 macrophages (Fulp et al., 2018). JC124 penetrated across the blood-brain barrier to reach brain tissues after oral administration. Moreover, it exhibited both in vitro and in vivo activities to ameliorate amyloid pathology and improve cognitive functions in a mouse model of Alzheimer’s disease, and it reduced neuroinflammatory responses following traumatic brain injury (Fulp et al., 2018; Yin et al., 2018; Kuwar et al., 2019). Therefore, benzenesulfonamides are potentially useful drug leads for developing therapeutics to treat acute and chronic neuronal injuries in the brain, such as Alzheimer’s disease, multiple sclerosis, and traumatic brain injury. JC124 also exhibited therapeutic activities toward acute myocardial infarction in two mouse models of infarction/reperfusion injury that used coronary artery ligation for 30 minutes and for 75 minutes, respectively; treatment with JC124 resulted in reduction of the infarct size and the serum troponin level (Fulp et al., 2018). Another derivative of sulfonamide, HL16, showed an improved inhibitory activity toward NLRP3 with an IC_50_ value of 1.3 *μ*M but it had reduced specificity as it inhibited NLRC4 and AIM2 in addition to inhibition of NLRP3. On the other hand, compound 17 (YQ128) selectively inhibited the NLRP3 inflammasome with an IC_50_ at 0.3 *μ*M and did not inhibit NLRC4 or AIM2 in J774A.1 macrophages (Jiang et al., 2019). Compound 17 penetrated across blood-brain barrier, which was more effective when administered by the intravenous than by the oral route, indicating a limited oral bioavailability.

## VI. Structural Insights into Inhibitor-NLRP3 Interaction

By using biophysical measurement, photoaffinity labeling-based analysis, crystallography, and computational modeling, several recent studies provided new insights into inhibitor-NLRP3 interaction at a molecular level.

### A. MCC950 and Derivatives

The finding that diarylsulfonylurea CRIDs are potent and specific inhibitors of IL-1*β* production from human monocytes in response to LPS and ATP is pivotal in the search for therapeutic drugs against IL-1*β*-driven inflammation (Perregaux et al., 2001). This inhibitory activity on IL-1*β* processing was independent of inhibition of ATP-activated K^+^ channels by sulfonylurea anti-diabetic drugs. Early efforts to identify the molecular targets of MCC950 led to the identification of glutathione S-transferase omega 1-1 (GSTO1-1) as a candidate target, as a ^14^C-labeled, epoxide-bearing CRID effectively binds to GSTO1-1 at its Cys32 residue (Laliberte et al., 2003). GSTO1-1 is unique among glutathione S-transferases in that it contains an active site cysteine residue but displays little activity with typical glutathione S-transferase substrates. Instead, GSTO1-1 exhibits a thiol transferase activity characteristic of glutaredoxin. The role of GSTO1-1 in the inhibition of IL-1β production by MCC950 remains elusive to this date. In a subsequent study, MCC950 was found to inhibit the NLRP3 inflammasome-mediated IL-1*β* production specifically and potently with an IC_50_ at ∼8 nM. Detailed characterization of the inhibition suggests NLRP3 itself or a protein closely linked to NLRP3 activation as the target for inhibition by MCC950 (Coll et al., 2015). Understanding how MCC950 interacts NLRP3 to inhibit the inflammasome soon became a highly desired but challenging task.

#### 1. Biophysical and Biochemical Evidence of MCC950-NLRP3 Binding

Biophysical and biochemical analyses were carried out in three separate studies to elucidate the interaction between MCC950 and NLRP3 at the molecular level. The findings revealed that MCC950 targets NLRP3 specifically by binding to its NACHT domain at the Walker regions, which inhibits ATP binding and/or ATP hydrolysis by its ATPase and promotes NLRP3 autoinhibition.

A bioluminescence resonance energy transfer–based proximity assay was used to probe intramolecular structural changes induced by MCC950. The study revealed that MCC950 binds NLRP3 directly and interferes with the conformational change associated with the opening of NLRP3 upon activation (Tapia-Abellán et al., 2019). Therefore, MCC950 promotes the preservation of the inactive conformation and, in the case of CAPS-associated gain-of-function mutants, induces the closure of an a priori open state. Molecular docking and dynamics simulation showed that MCC950 likely binds to the Walker B motif of NLRP3 NACHT. This notion was supported by mutations of the motif that prevented MCC950 from binding and inhibiting NLRP3.

Binding of MCC950 to NLRP3 NACHT was also demonstrated using a drug affinity responsive target stability approach where protein targets bound with a small molecule is protected from proteolysis (Coll et al., 2019). MCC950 protected NLRP3 without an activator or after priming by LPS and activation by nigericin. This finding implies that MCC950 binds to NLRP3 in its inactive as well as activated state. An MCC950-based, benzophenone and alkyne-containing photoaffinity probe was developed and was shown to inhibit NLRP3 activation by covalently cross-linking to NLRP3 upon activation by UV light. By using a combination of photoaffinity labeling, drug affinity responsive target stability, antibody epitope mapping, and deletion and site-specific mutations of NLRP3, MCC950 was shown to interact with the walker B motif within the NLRP3 NACHT domain directly, which inhibited ATP hydrolysis and consequently the activation of NLRP3 and formation of the NLRP3 inflammasome. In these scenarios, MCC950 did not compete with ATP for NLRP3 binding, which is consistent with the notion that ATP binding takes place at the Walker A motif of NLRP3 NACHT.

A third study used two different chemoproteomic strategies to probe MCC950-NLRP3 interactions (Vande Walle et al., 2019). In the first approach, photoaffinity labeling with a probe containing a photoreactive benzophenone group linked to MCC950 (i.e., PAL-CRID3) enabled the covalent labeling of target proteins of MCC950 in situ upon UV exposure. Separation of the proteins by SDS-PAGE allowed in gel fluorescence detection of the labeled protein adducts, which confirmed the labeling of NLRP3 by the probe. In the second approach, MCC950 was immobilized on polymers using the iBody technology and MCC950 targets recruited on the polymers were immunoprecipitated. iBody with immobilized MCC950 immunoprecipitated endogenous NLRP3 from LPS-primed, wild-type BMDMs. Deletion mutation studies showed that MCC950 bound to NACHT. Mutations of the Walker A motif in full-length NLRP3 abolished binding of the photoaffinity probe of MCC950, suggesting that an intact ATP-binding pocket is required for strong binding of MCC950 to NLRP3 NACHT.

#### 2. Crystal Structure of NLRP3 NACHT Bound With MCC950 Derivative

The crystal structure of recombinant NLRP3 NACHT bound with an ADP and a fluorescently labeled analog of MCC950 (i.e., NP3-301) provided structural insights into MCC950 binding to NLRP3 NACHT at a resolution of 2.85 Å (Dekker et al., 2021). Binding of the probe to NLRP3 was specific to NACHT with an EC_50_ of 46.5 nM. Binding was reversible and could be competed off by NP3-146, a close analog of MCC950. No binding was observed between the probe and PYD, NEK7, ASC, or Casp1. In this structure, the subdomains of NACHT (i.e., NBD, HD1, WHD, HD2), as well as the STAND elements (i.e., the Walker A, Sensor 1, and Walker B motives) are arranged like in other STAND NACHTs. The spatial arrangement leads to the formation of an inactive and closed conformation through interactions among several key residues, such as ADP-His522, Arg351-Glu527, and Arg262-Glu511 (Fig. 4). While ADP makes extensive contacts with the Walker A motif, NP3-146 binds in a newly formed inhibitor pocket that is shaped by all four NACHT subdomains and is close to the ADP binding site. NP3-146 makes crucial interactions with the main chain of Walker A by forming a hydrogen bond between Ala228 and its urea moiety (Fig. 4). It also interacts with Arg578 of HD2. The chloro-di-isopropylbenzene group of the inhibitor lies in a hydrophobic space formed by HD1, WHD, and HD2, whereas its sulfonyl group interacts with Arg351 of NBD. The fluorophore extends out and does not seem to interfere with protein binding.

The salt bridge between Sensor-I Arg351 and WHD Glu527 in NLRP NACHT is critical for the closed conformation of inactive NLRP3, as mutation Glu527Lys disrupts this interaction to cause CAPS phenotypes in humans. Binding with NP3-146 is predicted to disrupt this interaction between Arg351 and Glu527, but this is compensated by bridging to Arg578 of HD2 with additional interactions across other domains in the structure of NACHT bound with NP3-146. This NP3-146 induced stabilization appears to be stronger than that of the apo-stabilization mechanisms and therefore allows for strong inhibition of NLRP3. MCC950 has been shown to bind both the active and inactive NLRP3, and MCC950 is able to close the active conformation, particularly in the absence of MEK7. Therefore, it is rational to posit that both MCC950 and NP3-146 inhibit the activation of NLRP3 by stabilizing the closed, inactive conformation of monomeric NLRP3, which prevents the large conformational change necessary for the initial steps in inflammasome activation.

MCC950 analogs have the potential to be used for treating CAPS patients. Two NLRP3 mutants, Ala350Val and Leu351Pro, which are equivalent to Ala354 and Leu355 in the crystal structure, are known to cause CAPS. MCC950 effectively inhibited the wild-type and Ala350Val NLRP3 inflammasomes, but not the Leu351Pro inflammasome, in both the ex vivo mutant macrophages and knock-in mouse models (Vande Walle et al., 2019). In the crystal structure of NACHT, neither residue directly interacts with the bound inhibitor, but both are in a helix that positions Arg351 to interact with the sulfonylurea group of NP3-146. It is possible that Leu351Pro alters the positioning of Arg351 and thus weakens the binding of the inhibitor in the Leu351Pro mutant and diminishes substantially its inhibitory activity on the NLRP3 inflammasome.

The structures of NLRP3 bound with MCC950 or its analog obtained from the crystallization of NACHT and from biophysical and biochemical analyses of NLRP3 all support the binding of MCC950 to the NACHT domain. The exact location where MCC950 binds in NLRP3 NACHT, however, deviates among these structures with respect to the location of Walker A and Walker B motives. This discrepancy could be due to differences in the inhibitors, cell and protein preparations, and the methodology used, between crystals and solutions where inhibitor-protein interactions were observed, and among varied kinetics of inhibitor-NLRP3 binding in different testing systems. Further studies are needed to clarify this and other discrepancies on MCC950-NLRP3 binding from different studies and using different approaches.

#### 3. Binding of MCC950 in the Oligomeric Ring-Like Structures of Inactive NLRP3

As MCC950 binds NLRP3 with a high affinity and this binding stabilizes NLRP3, MCC950 is often included in the copurification of NLRP3 for structural analysis by cryo-EM. In the decamer structure of inactive human NLRP3, MCC950 (CRID3) binds to a cleft in NLRP3 formed by subdomains NBD, HD1, WHD, HD2, and trLRR near the nucleotide-binding pocket. The *K_D_* value of binding is 20 nM. The sulfonylurea group of MCC950 is localized on the backside of Walker A close to the side chains of Ala227 and Ala228 and is sandwiched between Arg351 and Arg578. Mutation of Ala228, Arg351, and Arg578 abolished MCC950 binding and NLRP3 activation by nigericin. Therefore, MCC950 interacts with residues from all subdomains to stabilize their conformation arrangement in inactive NLRP3. A hexameric ring structure of human NLRP3 lacking PYD but with an ADP and an MCC950 bound was obtained by cryo-EM (Ohto et al., 2022). In this structure, MCC950 bound to the bottom of the central cavity formed by all domains of the NLRP3 NACHT-LRR protein. NBD, HD2, and LRR formed the entrance and side walls of the cavity, whereas HD1 and WHT formed the bottom. MCC950 and ADP bound to NACHT in a proximity of ∼8 Å in distance. The sulfonyl amide moiety was near Walker A. The amide oxygen formed a hydrogen bond to Arg578 and the sulfonyl moiety formed an ionic contact with Arg351. Therefore, binding of MCC950 stabilizes the closed form of the NACHT characterized by ADP-mediated tight packing of the NACHT subdomains. Overall, the cryo-EM structures of MCC950-bound NLRP3 oligomers are consistent with the findings on the binding of MCC950 analogs to NACHT in the crystal structure of NACHT regarding its binding site in relation to Walker A and subdomains, as well as major binding determinants (Dekker et al., 2021).

### B. Non-MCC950-Based NLRP3 Inhibitors

A list of synthetic or plant-derived, non-MCC950-based, small chemical inhibitors of the NLRP3 inflammasome have been described. Although limited information is available to account for their mechanism of inhibition of NLRP3 in details, many drugs appear to target the ADP/ATP binding pocket within NLRP3 NACHT by binding to the domain reversibly or covalently.

#### 1. Reversible Binding to NACHT

Some inhibitors bind to NACHT reversibly in a manner analogous to MCC950 binding. CY-09 is an analog of small chemical inhibitor of CFTR, CFTR_(inh)_-172, without inhibitory activities on CFTR. CY-09 inhibited the NLRP3 inflammasome specifically both in vitro and in vivo (Jiang et al., 2017). To identify its targets, a biotinylated analog of CY-09 was used as an affinity reagent to pull down target proteins. CY-09 specifically pulled down the NLRP3 protein and its NACHT domain. Mutations of the Walker A motif, but not the Walker B motif, impaired the binding of CY-09 to NLRP3. Binding between CY-09 and purified NLRP3 exhibited a *K_D_* value of ∼500 nM. Binding of CY-09 to NLRP3 blocked the ATPase activity at a dose range of 0.1 and 1 *μ*M. These findings revealed that CY-09 binds to the Walker A motif of NLRP3 to abolish ATP binding and thereby blocks its ATPase activity and inflammasomal activation. Molecular docking confirmed that CY-09 docks readily into the ATP-binding pocket of NLRP3 NACHT.

C77 contains a pharmacophore of benzoxazolone acetamide identified by virtual screening to target the NLRP3 ATP/ADP binding site (Sebastian-Valverde et al., 2021). C77 and some other benzoxazolone acetamide derivatives inhibited the NLRP3 inflammasome and, to a lesser extent, the NLRC4 inflammasome, in microglial cells with high potencies. As expected, C77 inhibited the ATPase by binding to the NACHT ATP/ADP-binding pockets of NLRP3 and NLRC4. Several other drugs also target NLRP3 by binding to the NACHT domain and by inhibiting ATP/ADP binding and/or ATPase catalysis, though further studies are needed to elucidate the inhibitor-bound structures and their binding properties in details. These include tranilast (a tryptophan analog), MNS (a protein kinase inhibitor), and OLT1177 (an orally active *β*-sulfonyl nitril compound), all which inhibit NLRP3 with varying potencies and specificities. Compound 17 (YQ128) is a second-generation inhibitor of the NLRP3 inflammasome derived from benzenesulfonamide with high selectivity and potency and is a drug lead for treatment of acute and chronic CNS injuries and myocardial infarction. The molecular mechanism by which compound 17 inhibits NLRP3 is unclear. Because of its structural similarity to MCC950, it is assumed that compound 17 binds to NACHT and inhibits the ATP/ADP binding and ATPase activity in a similar manner to MCC950.

#### 2. Covalent Binding and S-alkylation

Oridonin is derived from herbal medicine *Rabdosia rubescens* and has strong anti-inflammatory activities. Oridonin inhibited the NLRP3 inflammasome in various models. A biotinylated oridonin was synthesized and was shown to pull down NLRP3, but not NLRP3 inflammasomal components NEK7, ASC, or Casp1, or other inflammasomal sensors including AIM2, NLRC4, or NLRP1, from lysates of primed BMDMs (He et al., 2018). Oridonin bound purified NLRP3 with a *K_D_* value of 52.5 nM. Further characterization of oridonin-NLRP3 binding revealed that binding was covalent and irreversible. Binding was abolished when a cysteine residue at 279 in the NACHT NBD domain was changed to alanine to create a Cys279Ala mutant of NLRP3. Binding was also lost when the α,β-carbon-carbon double bond of oridonin was reduced to a single bond. These results demonstrated that oridonin binds to Cys279 of NLRP3 NACHT via a Michael receptor reaction and S-alkylation leading to irreversible adduct formation on NLRP3.

Bay 11-7082 is a multifunctional phenyl vinyl sulfone that inhibits the NLRP3 inflammasome independently of its inhibitory activity on NF-*κ*B and IKK-*β* (Juliana et al., 2010). The compound inhibited NLRP3 ATPase, possibly by alkylating cysteine residues of NLRP3. In a similar manner, parthenolide, a sesquiterpene lactone alkylating agent, alkylated the NACHT domain and inhibited the ATPase of NLRP3 (Juliana et al., 2010). BOT-4-one, a benzoxathiole derivative, inhibited NLRP3 by alkylating NACHT and by inhibiting ATPase (Shim et al., 2017). Lastly, INF39, an acrylate derivative, is an irreversible inhibitor of NLRP3 (Cocco et al., 2017).

The value of covalent inhibitors of NLRP3 for clinical use in patients is sometimes questioned because of its irreversible nature and potential off-target effects, though some alkylating agents have been shown to be safe in the dose range for therapeutic activities in animals. Nonetheless, further studies are needed to ensure the concern on the safety of irreversible inhibitors of NLRP3 is addressed.

## VII. Conclusion and Perspective

The research on the NLRP3 inflammasome in the past 20 years has led to a rapid accumulation of a body of knowledge on its function, signaling, and structure, which has impacted our understanding of innate immune sensing and regulation of inflammation in health and disease to a significant degree. The recognition that aberrant NLRP3 activation and function is central to the initiation and propagation of chronic inflammation under a broad range of chronic conditions is particularly intriguing, as chronic, noncommunicable diseases associated with lifestyle change and aging have become a leading cause of mortality and disability in the world today. Fittingly, this rich body of information is being harnessed to guide development of therapeutics that are beneficial for treating various chronic illnesses. In this regard, the anti-IL-1*β* therapy with FDA-approved drugs anakinra, canakinumab, and rilonacept has proven to be efficacious in treating CAPS and rheumatoid arthritis but these protein-based drugs each have limitations. Targeting NLRP3 with small chemical inhibitors remains to be a direct and desirable approach to inhibition of the inflammasome with potentially better efficacy, reduced off-target effect, and improved pharmacokinetics and convenience for use. Pharmacological screening of synthetic or natural products and derivatives for inhibition of induced production and secretion of IL-1*β* from macrophages has been instrumental in identification of a list of small chemical inhibitors of NLRP3 activation with significant potency and specificity in vitro and in vivo. Among them, the diarylsulfonylureas, that is, MCC950 derivatives, hold a great promise, as they are highly potent and specific for inhibition of NLRP3 activation in a score of disease models. Drug safety is of increasing concern as some drug leads including MCC950 have shown certain toxicity in clinical trials. Designing better and safer inhibitors of NLRP3 for drug therapy becomes an eminent challenge that demands a better understanding of the mechanisms of activation and the structures of NLRP3 and its inflammasome.

A high-resolution structure of inactive monomeric NLRP3 bound with ADP and NEK7 was obtained by cryo-EM. The structure reveals an overall earring shape consisting of a globular NACHT domain and a curved LRR domain, both of which contact the C-lobe of NEK7 through three interfaces. Using NLRC4 as a guide, this structure was extended to model the active conformation and oligomerization of NLRP3. In this hypothetical NLRP3-NEK7 inflammasome, the NLRP3 NBD-HD1 module undergoes an approximately 90° rotation to allow the formation of a disc-like oligomer structure, which recruits ASCs via homotypic PYD-PYD interactions between NLRP3 and ASC. ASC in turn undertakes prion-like aggregation via its PYDs into fibrils, which recruits multiple pro-casp1 through CARD-CARD interactions between ASC and pro-casp1, leading to the autocleavage and activation of pro-casp1s. Inactive NLRP3 also exist as oligomers in ring-like or cage-like structures that are often associated with subcellular membrane structures. Formation of NLRP3 cages at the Golgi apparatus promotes dispersion of TGNs that transport NLRP3 oligomers for binding with nuclear NEK7 and activation of NLRP3. Activated NLRP3 oligomerizes to form PYD nucleation leads that direct the unidirectional, prion-like extension of ASCs into fibrous bundles, leading to the formation of ASC specks.

Binding of small chemical inhibitors to NLRP3 was revealed structurally for MCC950-NLRP3 interactions. Biophysical and photoaffinity labeling–based analyses revealed the NACHT Walker motives as the likely binding site for MCC950. A crystal structure of NLRP3 NACHT bound with an MCC950 analogy provided structural details of MCC950-NACHT interactions. In this crystal structure, the ADP-bound NACHT assumes an inactive conformation stabilized by several key interactions, including ADP-His522, Arg351-Glu527, and Arg262-Glu511 interactions. Licensing by NEK7 and activation by PAMPs and DAMPS allows large conformational changes to result in ATP/ADP exchange and ATP hydrolysis and, consequently, NACHT rotation and oligomerization of NLRP3. MCC950 analog NP3-146 binds NACHT in a newly formed inhibitor pocket close to the ADP binding site. NP3-146 makes several crucial contacts with NACHT, including an interaction with the main chain of Walker A through a hydrogen bond between Ala228 and its urea moiety. MCC950 binds both the active and inactive NLRP3 and likely inhibits NLRP3 activation by stabilizing the closed, inactive conformation of monomeric NLRP3. Together, this collection of structural findings on NLRP3 enables the depiction of key structural features of the inactive protein, as well as key steps for the licensing and activation of the inflammasome and its inhibition by small chemical inhibitors. This body of new information provides a molecular basis for the rational design and pharmacological evaluation of new generations of inhibitors of NLRP3 in near future, for the benefit of treating chronic inflammatory diseases that are rapidly increasing in prevalence in the world today.

## Abbreviations

AIM2: absent in melanoma 2
ASC: associated speck-like protein containing a CARD
BMDM: mouse bone marrow–derived macrophages
CAPS: cryopyrin-associated periodic syndromes
CARD: caspase-recruitment and activation domain
Casp1: caspase 1
CNS: central nervous system
CFTR: cystic fibrosis transmembrane conductance regulator
CRID: cytokine release inhibitory drug
DAMP: danger (damage) associated molecular pattern
DSS: dextran sodium sulfate
dTGN: dispersed *trans*-Golgi network
FDA: Food and Drug Administration
FISNA: fish-specific NACHT-associated domain
GSDMD: gasdermin D
HD: helical domain
IAV: influenza A virus
IL: interleukin
LRR: leucine-rich repeat
MSU: monosodium urate
MWS: Muckle-Wells syndrome
NACHT: domain present in NAIP, CIITA, HET-E, and TP1
NASH: nonalcoholic steatohepatitis
NBD: nucleotide-binding domain
NEK7: never in mitosis gene A–related kinase 7
NLR: Nod-like receptor
NLRC4: NLR family CARD domain-containing protein 4
NLRP3: nucleotide-binding, oligomerization domain-like receptor family pyrin domain containing 3
NOMID: neonatal-onset multisystem inflammatory disorder
NF-*κ*B: nuclear factor kappa B
oxPAPC: oxidized phospholipid 1-palmitoyl-2-arachinonyl-sn-glycero-3-phosphorylcholine
P2X_7_: P2X purinoceptor 7
PAMP: pathogen associated molecular pattern
PARP1: poly(ADP-ribose) polymerase 1
PYD: pyrin domain
ROS: reactive oxygen species
STAND: signal transduction ATPases with numerous domains
TLR: toll-like receptors
TXNIP: thioredoxin interacting protein
WHD: winged helix domain
